# Mitochondrial Quality Control in Health and Disease

**DOI:** 10.1002/mco2.70319

**Published:** 2025-08-15

**Authors:** Lin Ye, Xinzhi Fu, Qi Li

**Affiliations:** ^1^ School of Basic Medicine Qingdao University Qingdao Shandong China; ^2^ School of Nursing Qingdao University Qingdao Shandong China

**Keywords:** disease intervention, mitochondria, mitochondrial quality control, therapeutic strategies

## Abstract

Mitochondria are central regulators of cellular energy metabolism, and their functional integrity is essential for maintaining cellular homeostasis. Mitochondrial quality control (MQC) encompasses a coordinated network of mitochondrial biogenesis, dynamics (fusion and fission), and selective autophagy (mitophagy), which together sustain mitochondrial structure and function. Under physiological conditions, MQC ensures the removal of dysfunctional mitochondria, restricts excessive reactive oxygen species production, and modulates apoptosis, thereby supporting the high energy demands of organs such as the heart and brain. Disruption of MQC contributes to the onset and progression of various diseases, including neurodegenerative disorders, cardiovascular pathologies, and metabolic syndromes, largely through accumulation of damaged mitochondria and impaired metabolic signaling. While the core components of MQC have been characterized, the mechanistic interplay among its modules and their disease‐specific alterations remain incompletely defined. This review provides an integrated overview of the molecular pathways governing mitochondrial biogenesis, dynamics, and mitophagy, with a focus on their cross‐talk in maintaining mitochondrial homeostasis. We further discuss how MQC dysfunction contributes to disease pathogenesis and examine emerging therapeutic approaches aimed at restoring mitochondrial quality. Understanding the regulatory logic of MQC not only elucidates fundamental principles of cellular stress adaptation but also informs novel strategies for disease intervention.

## Graphical abstract

Mitochondrial quality control (MQC) maintains cellular homeostasis by coordinating various processes such as fission, fusion, mitophagy, and mitochondria‐regulated cell death. Dysregulation of these mechanisms plays a key role in the development of many human diseases. Interventions targeting different components of MQC offer potential therapeutic avenues for treating various diseases, including inhibiting mitochondrial fission, enhancing mitochondrial fusion, promoting moderate mitophagy, and attenuating mitochondria‐mediated cell death pathways.

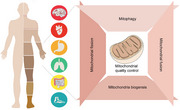

## 1 Introduction

Mitochondria are central to cellular energy metabolism, generating adenosine triphosphate (ATP) through oxidative phosphorylation (OXPHOS) to support the energy demands of high‐metabolic tissues such as the heart and brain [1, 2, 3]. In addition to ATP production, the electron transport chain (ETC) is a primary source of reactive oxygen species (ROS). While moderate levels of ROS act as signaling molecules, excessive ROS can induce oxidative damage to mitochondrial DNA (mtDNA), proteins, and lipids, thereby impairing mitochondrial function [4, 5]. Environmental stressors, including hypoxia, nutrient imbalance, and toxins, further exacerbate mitochondrial vulnerability. Beyond energy metabolism, mitochondria are key regulators of apoptosis. They initiate intrinsic cell death pathways through the release of proapoptotic factors such as cytochrome *c*, linking mitochondrial integrity directly to cell fate decisions. Thus, the functional maintenance of mitochondria is not only essential for sustaining cellular energy homeostasis but also for regulating survival signaling under both physiological and pathological conditions.

**FIGURE 1:**
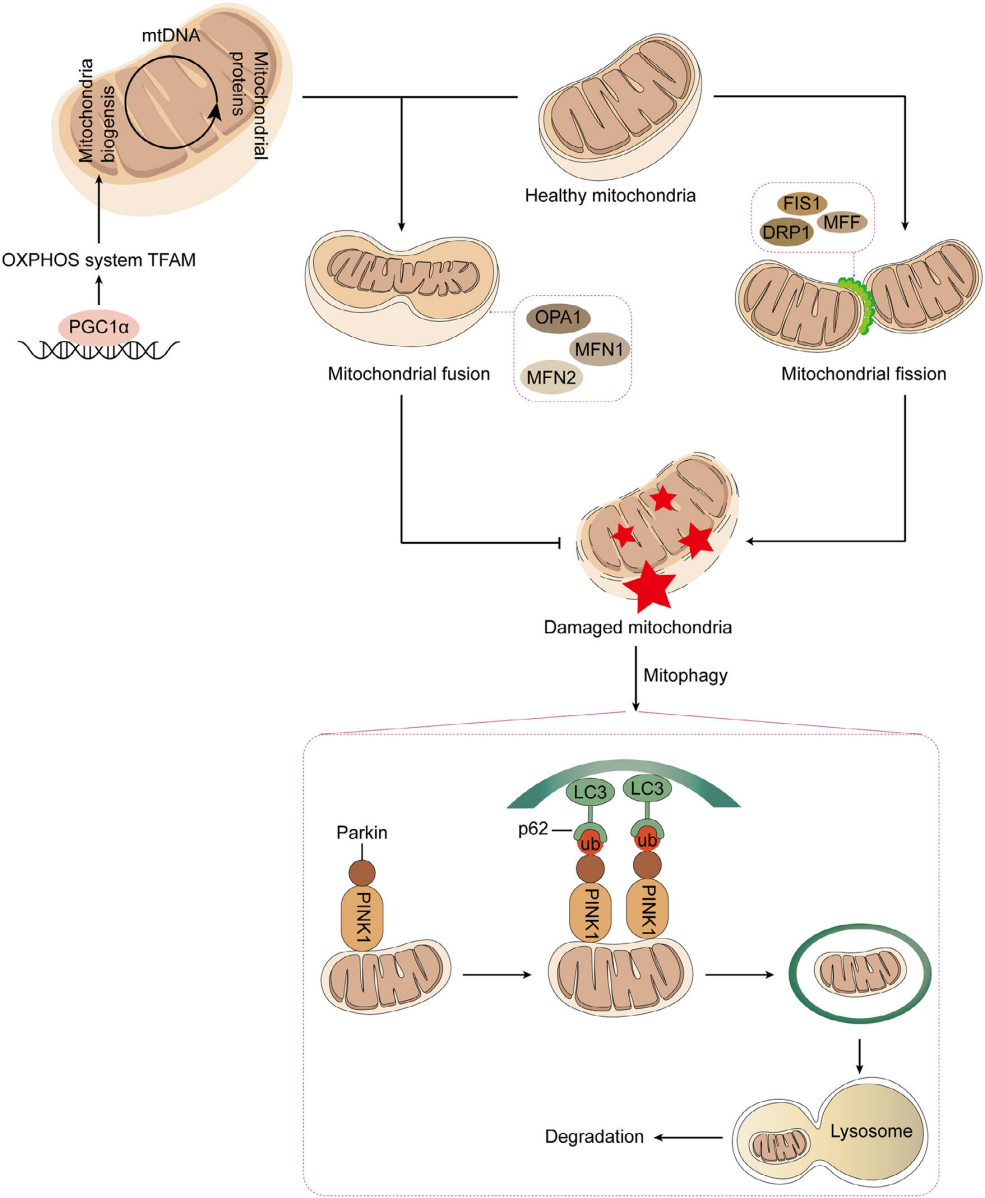
Mitochondrial quality control. The MQC system constitutes a multilayered regulatory network essential for preserving mitochondrial integrity and functional homeostasis. This system orchestrates a series of interconnected processes—including mitochondrial biogenesis, fission, fusion, protein degradation, and mitophagy—to maintain a healthy mitochondrial population within the cell. Over time, mitochondrial function tends to decline; under such conditions, fusion events mediated by proteins such as OPA1, MFN1, and MFN2 enable the exchange of intramitochondrial components, thereby sustaining mitochondrial performance. In contrast, persistent mitochondrial dysfunction activates fission machinery involving FIS1, DRP1, and MFF, which facilitates the segregation and elimination of damaged organelles. During this process, relatively intact mitochondria are reintegrated into the network via continued fusion, whereas impaired mitochondria are selectively removed through mitophagy, predominantly via the canonical PINK1–Parkin pathway. The dynamic interplay among fusion, fission, and mitophagy ensures optimal mitochondrial turnover and prepares the cell for the demands of mitochondrial biogenesis. Mitochondrial biogenesis, a critical regenerative mechanism, serves to replenish the mitochondrial pool by replacing senescent or damaged mitochondria. This process is tightly regulated by both mtDNA and nDNA, and is governed by transcriptional coactivators and regulators such as PGC‐1α, which drive the expression of genes involved in mitochondrial replication and oxidative metabolism. Newly synthesized mitochondria resulting from biogenesis enhance ATP production, thereby supporting cellular metabolic needs under both physiological and pathological conditions. *Abbreviations*: ATP, adenosine triphosphate; Drp1, dynamin‐related protein 1; FIS1, fission mitochondrial 1; MFF, mitochondrial fission factor; MFN1, mitofusin‐1; MFN2, mitofusin‐2; MQC, mitochondrial quality control; mtDNA, mitochondrial DNA; nDNA, nuclear DNA; OPA1, optic atrophy 1; PGC‐1α, peroxisome proliferators‐activated receptor γ coactivator l alpha; PINK1, PTEN‐induced putative kinase 1.

To cope with increased metabolic demand and stress, cells rely on a mitochondrial quality control (MQC) system that safeguards mitochondrial integrity. MQC comprises a set of interrelated processes—including mitochondrial biogenesis, dynamic remodeling (fusion and fission), mitophagy, and protein/DNA repair—that operate in concert to preserve mitochondrial structure and function. *Mitochondrial biogenesis*: This process involves the continuous synthesis of new mitochondria to replace aging or damaged ones. It includes the coordinated assembly of mitochondrial inner and outer membranes as well as matrix components, ensuring the maintenance of mitochondrial quantity and functionality. *Mitochondrial repair*: Repair mechanisms address damage to the mitochondrial inner membrane, correct DNA lesions, and degrade dysfunctional proteins. These processes are crucial for restoring the operational capacity of compromised mitochondria. *Quality control*: Cells employ specialized pathways to detect and eliminate defective mitochondria. For instance, mitophagy selectively identifies and degrades damaged mitochondria, while cytosolic protein tagging further facilitates the clearance of dysfunctional organelles. *mtDNA maintenance*: Given that mitochondria contain their own genome encoding essential proteins, cells must preserve and repair mtDNA to ensure the stability and continuity of mitochondrial function (Figure 1). Disruption of any MQC mechanism can lead to mitochondrial dysfunction, subsequently compromising overall cellular and tissue health. Therefore, the proper operation of the MQC system is not only vital for maintaining mitochondrial homeostasis but also plays a crucial role in enabling cells to adapt to environmental stressors and pathological conditions.

This review provides a comprehensive analysis of the mechanisms underlying MQC, with an emphasis on mitochondrial biogenesis, dynamics, and mitophagy. We discuss their integrative roles in health maintenance and their dysregulation in pathological contexts, including ischemia–reperfusion (I/R) injury (IRI), neurodegenerative disorders, and chronic inflammation. Finally, we highlight emerging therapeutic strategies targeting MQC, offering new perspectives for disease intervention and mitochondrial protection.

## 2 Mechanisms of MQC

### 2.1 Mitochondrial Biogenesis

Mitochondrial biogenesis is a fundamental component of the MQC system, essential for maintaining mitochondrial functionality and cellular homeostasis [6, 7, 8]. It involves both the de novo synthesis and expansion of pre‐existing mitochondria to ensure adequate organelle number and efficiency in response to metabolic demands. This process is tightly regulated by a network of transcriptional and posttranscriptional mechanisms [6]. Central to the regulation of mitochondrial biogenesis are several transcriptional activators and coactivators, including peroxisome proliferator‐activated receptor gamma coactivator‐1 alpha (PGC‐1α), nuclear respiratory factors 1 and 2 (NRF1/NRF2), and peroxisome proliferator‐activated receptor alpha (PPAR‐α). These factors respond to cellular energy status, redox changes, and environmental cues to activate genes involved in mtDNA replication, mitochondrial protein synthesis, and respiratory chain assembly. PGC‐1α, a master regulator of mitochondrial biogenesis, is predominantly expressed in metabolically active tissues such as the heart, brain, kidney, and brown adipose tissue [9, 10, 11, 12]. Initially, PGC‐1α was characterized as a cold‐inducible coactivator that regulates adaptive thermogenesis in BAT and skeletal muscle by stimulating mitochondrial biogenesis and oxidative metabolism [13]. Initially identified as a cold‐inducible factor promoting thermogenesis in brown fat and skeletal muscle, PGC‐1α has since been shown to modulate mitochondrial function across various physiological and pathological contexts. For instance, within the tumor microenvironment, cancer‐associated fibroblasts upregulate the expression, nuclear localization, and transcriptional activity of PGC‐1α, thereby promoting mitochondrial biogenesis and tumor cell migration. Conversely, shikonin facilitates the phosphorylation of PGC‐1α at the Thr‐295 residue, which enhances its interaction with NEDD4 E3 ubiquitin protein ligase (NEDD4), leading to ubiquitin‐mediated degradation. This degradation disrupts the PGC‐1α/ERRα axis and downregulates downstream mitochondrial gene expression, thus inhibiting mitochondrial biogenesis and tumor metastasis [14]. Similarly, during myoblast differentiation, PGC‐1α mediates mitochondrial regeneration via PGC1‐α and upregulates the fusion protein optic atrophy 1 (OPA1) to restore a healthy mitochondrial network. This process is intimately linked with the activation of early autophagy, underscoring the crucial role of autophagy in maintaining mitochondrial quality and function [15]. In addition to these processes, oxygen availability further regulates mitochondrial biogenesis via posttranslational modifications of PGC‐1α. Under normoxic conditions, lysine demethylase 3A (KDM3A) demethylates the K‐224 residue of PGC‐1α, thereby enhancing its interaction with Nrf1/Nrf2 and promoting the transcription of mitochondrial genes. In contrast, hypoxia inhibits KDM3A activity, resulting in the mono‐methylation of PGC‐1α at K‐224, which diminishes its transcriptional activity [16]. Notably, in brown adipocytes, cardiotrophin like cytokine factor 1 activates the ciliary neurotrophic factor receptor/signal transducer and activator of transcription 3 (CNTFR/STAT3) signaling pathway, leading to the transcriptional repression of PGC‐1α and PGC‐1β, consequently suppressing mitochondrial biogenesis and thermogenic function [17]. In the context of acute kidney injury, aldehyde dehydrogenase 2 family member promotes the nuclear translocation and activation of PGC‐1α, thereby enhancing mitochondrial function and mitigating renal damage [18]. In conclusion, mitochondrial biogenesis and quality control collaborate to sustain cellular energy balance. As a pivotal regulator, PGC‐1α orchestrates diverse pathways to regulate mitochondrial activity, providing promising targets for exploring mitochondrial‐associated disorders.

### 2.2 Regulation of Mitochondrial Protein Homeostasis

Mitochondrial protein synthesis and trafficking are fundamental to the maintenance of organelle integrity and function. Uniquely, the vast majority of mitochondrial proteins are encoded by nuclear DNA, synthesized on cytosolic ribosomes, and subsequently imported into mitochondria in a process termed mitochondrial protein biogenesis. These precursor proteins contain mitochondrial targeting sequences that facilitate their accurate delivery to specific mitochondrial compartments, ensuring appropriate localization and functional integration. This multistep process requires precise coordination of transcription, translation, translocation, folding, and insertion, and is under tight quality control. This process is not only crucial for the proper maintenance of mitochondrial function but also significantly impacts overall cellular homeostasis. First, errors during the transcription of mtDNA can lead to the production of defective mRNAs, and such errors are amplified during translation, potentially resulting in misfolded nascent polypeptides and functional abnormalities. Studies indicate that mitochondrial RNA polymerase (POLRMT) exhibits a relatively high error rate during transcription [19, 20]. Although the transcription elongation factor TEFM extends polycistronic transcripts, it may also introduce mutations [21]. These incomplete or aberrantly modified mRNAs often escape effective surveillance mechanisms, leading to the production of fusion open reading frames and misfolded proteins during translation, thereby jeopardizing mitochondrial proteostasis. Second, mitochondrial ribosomes function beyond polypeptide elongation by acting as central quality control hubs. When mitochondrial mRNAs lack stop codons or encounter other translational impediments, release factors such as MTRFR mediate translation termination, preventing ribosomal stalling and the accumulation of aberrant translation products [22]. Concurrently, misfolded nascent chains are promptly recognized and degraded by membrane‐bound protease complexes, such as AFG3 like matrix AAA peptidase subunit 2, thereby averting the accumulation of aberrant polypeptides within the membrane that could trigger stress responses [23]. When severe defects arise during mitochondrial protein synthesis, a cascade of stress responses is triggered. For instance, the accumulation of misfolded nascent polypeptides can activate the OMA1 zinc metallopeptidase (OMA1) protease, leading to remodeling of the mitochondrial inner membrane and alterations in membrane dynamics [24, 25]. This activation may further initiate ribosome and mRNA degradation pathways, ultimately reducing the capacity for mitochondrial protein synthesis and compromising the stability of OXPHOS complexes. Overall, mitochondrial protein synthesis and its quality control mechanisms employ multilayered regulatory processes to ensure the proper folding, localization, and functionality of proteins. In‐depth investigation of these mechanisms not only aids in unraveling the molecular basis of mitochondrial dysfunction but also provides potential targets and theoretical foundations for developing therapeutic strategies against mitochondrial‐related diseases.

### 2.3 Mitochondrial Dynamics

#### 2.3.1 Mitochondrial Fission

Mitochondrial fission is a dynamic process that involves the division of mitochondria into smaller units and is a critical aspect of mitochondrial dynamics [26]. This process not only increases the number of mitochondria within a cell but also modulates their morphology and function. Under normal physiological conditions, mitochondrial fission helps to meet the energy demands of various cellular regions and is involved in the repair and renewal of damaged mitochondria [27]. During cell division, mitochondrial fission ensures the proper distribution of mitochondria into daughter cells, thereby maintaining cellular metabolism and energy requirements. In response to injury or other pathological states, mitochondrial fission facilitates the segregation and degradation of damaged portions, thereby preserving the health and functionality of mitochondria and ensuring the physiological functions and homeostasis of the cell [6, 28, 29].

In this process, dynamin‐related protein 1 (Drp1), also known as dynamin‐like protein 1 (DNML1), is a key regulatory factor of mitochondrial fission and also plays a crucial role in maintaining MQC [30]. Drp1, a member of the dynamin protein family, is a GTPase that translocates from the cytoplasm to the mitochondria in response to cellular signals [31]. Upon reaching the mitochondrial surface, Drp1 anchors to the outer mitochondrial membrane through adaptor proteins, including fission, mitochondrial 1 (Fis1), mitochondrial fission factor (MFF), mitochondrial elongation factor 2 (MID49), and MID51. It then assembles into helical oligomers, where it uses GTP hydrolysis to facilitate mitochondrial constriction and division [30, 32, 33, 34, 35, 36]. For example, under conditions that require mitochondrial fission, DRP1 is recruited to the mitochondria, initiating the fission process [26, 37]. The activity of Drp1 is tightly regulated by multiple factors, including posttranslational modifications [38, 39, 40, 41], actin polymerization [42, 43, 44], and interactions with other organelles such as the endoplasmic reticulum (ER), Golgi apparatus, and lysosomes [26, 44, 45, 46]. Drp1‐mediated mitochondrial fission plays a vital role in MQC. On one hand, by promoting asymmetric fission, damaged mitochondrial components are segregated from healthy mitochondria, resulting in the formation of a functional mitochondrial fragment and a dysfunctional one [31]. The latter is then marked for mitophagy, which ensures the clearance of damaged mitochondria, maintaining the overall health and function of the mitochondrial network [47]. For example, under pathological conditions with mitochondrial damage, Drp1 triggers asymmetric fission to promote the elimination of dysfunctional mitochondria [31]. On the other hand, Drp1 is essential for maintaining mitochondrial function and quality in specific cell types, such as cardiomyocytes. Studies in cardiomyocyte‐specific Drp1 knockout mice reveal mitochondrial dysfunction and cardiac disease, underscoring the critical role of Drp1 in maintaining mitochondrial quality within heart cells [48].

#### 2.3.2 Mitochondrial Fusion

Mitochondrial fusion is a critical process in mitochondrial dynamics, working in concert with mitochondrial fission to maintain mitochondrial morphology and function [49]. This process involves the fusion of the outer membranes of two or more mitochondria, forming larger mitochondrial structures, typically in response to increased cellular energy demand or stress [50, 51]. Mitochondrial fusion helps restore mitochondrial function, facilitates mitochondrial genome exchange, and plays a protective role during cellular stress, thereby maintaining the integrity of the mitochondrial network. The key regulatory proteins involved in mitochondrial fusion include Mitofusin1 (Mfn1), Mitofusin2 (Mfn2), and OPA1 [51]. Mfn1 and Mfn2 are located on the outer mitochondrial membrane, where they mediate outer membrane fusion, while OPA1 is found on the inner mitochondrial membrane and regulates inner membrane fusion [52, 53, 54, 55, 56, 57]. Through the coordinated action of these fusion factors, mitochondria can fuse at appropriate times to preserve mitochondrial functional stability, ensuring efficient energy supply and cellular stress adaptation. Research has identified mitochondrial fission process 1 (MTFP1) as a negative regulator of mitochondrial fusion, which inhibits OPA1 oligomerization by altering mitochondrial membrane curvature and lipid distribution, thereby reducing mitochondrial fusion. The absence of MTFP1 results in increased mitochondrial fusion, which improves mitochondrial abnormalities by modulating mtDNA copy number and participating in MQC [58]. In hepatocytes, OPA1, a key protein in mitochondrial fusion, is essential for maintaining metabolic homeostasis; its deficiency not only inhibits the development of obesity and metabolic disorders but also disrupts the interaction between mitochondria and other organelles, thereby affecting bile acid synthesis and lipid absorption [59]. In platelets and neural cells, MFN1 and MFN2 are critical for mediating mitochondrial fusion, and their deletion leads to mitochondrial structural damage, loss of cellular function, and impacts the progression of various diseases [60]. Specifically, MFN2 in platelets is crucial for maintaining mitochondrial integrity, platelet lifespan, and hemostatic function, highlighting the vital role of mitochondrial fusion in cellular function within both the hematological and nervous systems [61]. These findings underscore the importance of mitochondrial fusion in maintaining cellular homeostasis and its potential as a therapeutic target in various metabolic, neurological, and hematological disorders.

### 2.4 Mitophagy

Mitophagy is a mechanism by which cells selectively degrade surplus or defective mitochondria through the autophagy pathway. Its purpose is to maintain the quality and quantity of mitochondria within the cell [62, 63, 64]. This process clears unwanted mitochondria during development and adjusts the number of mitochondria based on changing metabolic demands [65]. Mitophagy also serves as a crucial component of MQC, identifying and tagging severely damaged mitochondria for timely removal. Defects in mitophagy have been associated with various human diseases [65, 66]. The mechanism of mitophagy involves two main pathways, namely, the phosphatase and tensin homolog (PTEN)‐induced kinase 1/parkin RBR E3 ubiquitin protein ligase (PINK1/Parkin) pathway and the BCL2 interacting protein 3 (BNIP3) pathway. These pathways recognize and eliminate damaged mitochondria through different mechanisms [67]. *PINK1–Parkin pathway*: PINK1 is a mitochondrial protein kinase that is normally imported into mitochondria and rapidly degraded. However, when mitochondria are damaged or depolarized, the import of PINK1 is blocked, leading to its accumulation on the outer mitochondrial membrane. This activates the protein Parkin, which is a cytoplasmic ubiquitin ligase. Activated Parkin builds polyubiquitin chains on damaged mitochondria, recruiting receptor proteins such as nuclear dot protein 52 (NDP52) and optineurin. These receptors interact with microtubule‐associated protein 1 light chain 3 (LC3B) on the autophagosome, promoting the engulfment of mitochondria into autophagosomes, which are then degraded [67, 68, 69, 70, 71, 72, 73]. Studies have demonstrated that PINK1–Parkin‐mediated mitophagy effectively removes damaged mitochondria and excess ROS, thereby maintaining mitochondrial homeostasis within cells. Further research identified that polymerized porcine hemoglobin (pPolyHb), a novel hemoglobin‐based oxygen carrier, exerts significant protective effects against IRI in H9c2 cardiomyocytes by modulating the PINK1–Parkin pathway [74]. Specifically, pPolyHb was shown to significantly reduce apoptosis rates and enhance the survival of H9c2 cells. Additionally, pPolyHb gradually decreased the expression levels of PINK1 and Parkin during the reperfusion phase. Moreover, another study highlighted the critical role of Notch1 in mitigating myocardial IRI by regulating mitochondrial function. Notch1 was found to alleviate mitochondrial dysfunction and fragmentation by inhibiting the PTEN–PINK1 pathway, thereby conferring cardioprotection in the context of IRI [75]. These findings underscore the importance of targeting the PINK1‐Parkin signaling axis as a therapeutic strategy to reduce myocardial damage and preserve mitochondrial stability. *Mitophagy receptor pathway*: This pathway involves multiple receptor proteins such as BNIP3, BCL2 interacting protein 3 like, FUN14 domain containing 1 (FUNDC1), and SMAD‐specific E3 ubiquitin protein ligase 1, which are recruited to damaged mitochondria and guide them to autophagosomes for degradation by interacting with LC3 on the auto‐phagosomal membrane [66, 76, 77, 78, 79, 80]. To elucidate the in vivo role of mitophagy, researchers generated a FUNDC1 knockout mouse model and employed genetic and biochemical approaches, including the use of synthetic peptides to disrupt the FUNDC1–LC3 interaction. The findings revealed that mitophagy plays a critical role in regulating mitochondrial quantity and quality under hypoxic conditions induced by IRI. Moreover, hypoxic preconditioning was shown to activate FUNDC1‐dependent mitophagy in platelets, effectively modulating platelet activity and mitigating I/R‐induced cardiac damage [81]. These results highlight the protective potential of FUNDC1‐mediated mitophagy in maintaining cardiovascular health.

## 3 MQC in Major Diseases

Mitochondria, as essential organelles in eukaryotic cells, play a central role in maintaining cellular homeostasis and functionality [2]. They are not only pivotal pathological drivers in the onset and progression of numerous diseases but also serve as critical diagnostic biomarkers, demonstrating dual significance [82, 83, 84, 85]. On one hand, mitochondrial dysfunction directly contributes to the pathogenesis of various conditions by disrupting cellular energy metabolism, redox balance, and signaling pathways [86]. On the other hand, the dynamic alterations in mitochondrial morphology, biogenesis, and metabolic activity provide molecular‐level insights for disease diagnosis and monitoring [87]. This unique characteristic of mitochondria, being “both the cause and consequence” of pathological processes, underscores their critical importance as a focal point in disease research. MQC, as a fundamental mechanism supporting mitochondrial function and maintaining cellular homeostasis, plays a critical role in regulating mitochondrial dynamics, metabolic processes, and protein homeostasis. Increasing evidence has demonstrated that dysregulation of MQC is closely associated with the pathogenesis of numerous major diseases, including ischemia–hypoxia‐related conditions, inflammatory diseases, and neurodegenerative diseases. Pathological changes resulting from MQC imbalance are primarily characterized by abnormalities in mitochondrial morphology and dynamics, metabolic dysfunctions, impaired mitophagy, and disturbances in protein homeostasis. These dysfunctions collectively contribute to cellular damage, oxidative stress, and inflammatory responses, ultimately driving disease onset and progression. A deeper understanding of MQC's role in these diseases underscores its significance as a critical target for elucidating disease mechanisms and developing precise therapeutic interventions. Research into MQC not only provides profound insights into the molecular pathophysiology of major diseases but also offers innovative strategies for advancing diagnostic and therapeutic approaches, highlighting its potential to reshape disease management and improve clinical outcomes.

### 3.1 Ischemia–Hypoxia‐Associated Diseases

#### 3.1.1 Brain Ischemia/Hypoxia

**FIGURE 2:**
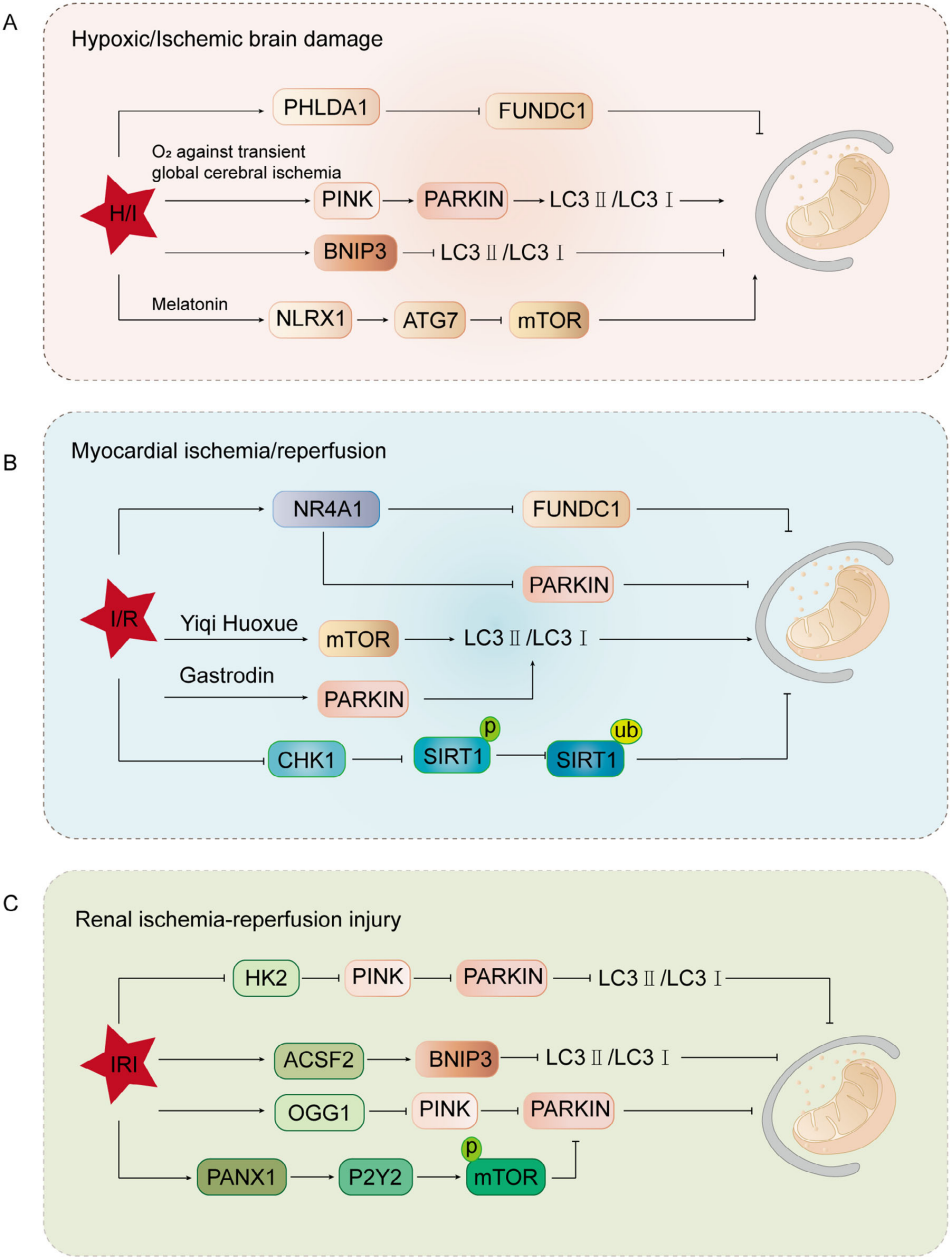
The role of mitochondrial autophagy in ischemia–hypoxia‐associated diseases. (A) In HI brain injury, PHLDA1 exacerbates brain damage by suppressing FUNDC1‐mediated mitophagy. HPC with 8% O_2_ alleviates hypoxic–ischemic brain injury following tGCI by activating the PINK1/Parkin pathway to promote mitophagy. In HI brain injury, activation of the BNIP3‐mediated mitophagy pathway regulates iron homeostasis and redox balance, thereby inhibiting the occurrence of ferroptosis. In HI brain injury, melatonin regulates mitophagy by upregulating the expression of NLRX1, Beclin‐1, and ATG7, and downregulating the expression of mTOR, thereby protecting neuronal cells from damage. (B) NR4A1 negatively regulates myocardial ischemia–reperfusion injury by activating Mff‐mediated mitochondrial fission and inhibiting FUNDC1‐mediated mitophagy. Additionally, NR4A1 also suppresses Parkin‐driven mitophagy, leading to impaired cardiac function. CHK1 maintains mitochondrial homeostasis and mitophagy by phosphorylating SIRT1 and inhibiting SMURF2‐mediated degradation, thereby protecting the heart from I/R injury through antioxidative stress and promoting mitochondrial biogenesis. Yiqi Huoxue alleviates I/R injury by regulating mitophagy, improving mitochondrial structure and function, and reducing oxidative stress. Gastrodin improves mitophagy by activating the PINK1/Parkin pathway, thereby reducing myocardial I/R injury. (C) In IRI, HIF‐1α CTAD transcriptionally regulates HK2 and activates PINK1/Parkin‐mediated mitophagy, thereby alleviating kidney injury. Inhibiting ACSF2 enhances IRI‐induced mitophagy, restores mitochondrial function, and further promotes mitophagy by regulating BNIP3, thereby alleviating IRI. OGG1 negatively regulates mitophagy by suppressing the PINK1/Parkin pathway in IRI, thereby exacerbating IRI. PANX1 exacerbates IRI by regulating the ATP–P2Y–mTOR signaling pathway to inhibit mitophagy. *Abbreviations*: HPC, hypoxic postconditioning; tGCI, transient global cerebral ischemia; PINK1, PTEN‐induced putative kinase 1; BNIP3, BCL2 interacting protein 3; NLRX1, NLR family member X1; ATG7, autophagy related 7; mTOR, mammalian target of rapamycin; NR4A1, nuclear receptor 4A1; Mff, mitochondrial fission factor; FUNDC1, FUN14 domain containing 1; CHK1, checkpoint kinase 1; SIRT1, silent information regulator 1; SMURF2, SMAD‐specific E3 ubiquitin protein ligase 2; CTAD, C‐terminal transactivation domain; HK2, hexokinase 2; ACSF2, acyl‐CoA synthetase family member 2; OGG1, 8‐oxoguanine DNA glycosylase‐1; PANX1, Pannexin1.

Brain ischemia–hypoxia is a pathological condition caused by reduced cerebral blood flow or insufficient oxygen supply, leading to a significant decrease in energy availability within brain tissues and severely impairing cellular functionality and viability [88]. Under ischemic and hypoxic conditions, cellular metabolism shifts from aerobic to anaerobic pathways, accompanied by ATP depletion and lactate accumulation, triggering a cascade of pathological responses including excitotoxicity, lipid peroxidation, and oxidative stress. Furthermore, prolonged ischemia and hypoxia activate apoptotic and necrotic pathways, resulting in irreversible damage to brain cells [89, 90, 91]. In this process, mitochondria, as the central hub of cellular energy metabolism, play a critical role, and their dysfunction directly leads to ATP depletion and exacerbates oxidative stress [84]. Therefore, maintaining MQC is essential for stabilizing mitochondrial function and alleviating oxidative stress. Mitophagy, as a selective mechanism for clearing damaged mitochondria, helps alleviate neuronal injury. In the neonatal hypoxia–ischemia (HI) rat model and OGD/R‐treated primary hippocampal neurons, the expression of Pleckstrin Homology Like Domain Family A Member 1 (PHLDA1) was significantly upregulated. Mechanistically, knockdown of PHLDA1 enhanced mitophagy by activating FUNDC1, which alleviated neuronal injury and improved cognitive function. However, knockdown of FUNDC1 or pretreatment with mitochondrial division inhibitor‐1 (Mdivi‐1) inhibited mitophagy, increased brain infarct volume, and reversed the neuroprotective effects of PHLDA1 knockdown. These findings suggest that PHLDA1 exacerbates HI‐induced brain injury by inhibiting FUNDC1‐mediated mitophagy [92]. Mitophagy plays a crucial role in transient global cerebral ischemia (tGCI) tolerance. Studies have shown that, in the later stages of tGCI reperfusion, 8% O_2_ hypoxia preconditioning (HPC) reduces the accumulation of mitochondrial‐associated proteins in the Cornu Ammonis 1 region and increases the LC3II/I ratio, promoting mitophagosome formation. Mechanistically, HPC activates the PINK1/Parkin pathway, enhancing mitochondrial levels of PINK1 and Parkin, as well as their ubiquitination, thereby boosting mitophagy and alleviating neuronal damage. These findings suggest that HPC confers neuroprotection against tGCI by activating the PINK1/Parkin‐dependent mitophagy pathway [93]. Recent studies have shown that hypoxic–ischemic brain damage (HIBD) induces ferroptosis in neurons. Ferroptosis inhibitors such as ferrostatin‐1 and deferoxamine B can reverse the accumulation of iron and lipid peroxides induced by Mdivi‐1, thereby alleviating ferroptosis triggered by HI. Mechanistically, BNIP3‐mediated mitophagy plays a crucial role by activating the sequestosome 1‐Kelch like ECH‐associated protein 1‐Nrf2 (P62–KEAP1–Nrf2) pathway, which regulates iron and redox homeostasis, thus enhancing cellular resistance to ferroptosis. Notably, BNIP3‐mediated mitophagy does not mitigate neuronal ferroptosis via the glutathione peroxidase 4–glutathione (GPX4–GSH) axis but rather by modulating key factors involved in iron and redox balance. This finding provides new insights into potential therapeutic strategies for HIBD [94]. In summary, activation of mitophagy is an important pathway for preventing HIBD. Researchers have found that melatonin (MT) exerts neuroprotective effects in HIBD by modulating mitophagy. Specifically, MT upregulates the expression of NLRX1, Beclin‐1, and autophagy‐related protein 7 (ATG7), while downregulating the expression of mammalian target of rapamycin (mTOR) and mitochondrial inner membrane translocase 23. NLRX1 plays a crucial role in mediating MT‐induced neuroprotection by regulating mitophagy (Figure 2A) [95].

In addition, mitochondrial dynamics, as another aspect of MQC, also plays a crucial role in HIBD. In oxygen‐glucose deprivation (OGD)‐treated neuronal cells and ischemic brain tissues induced by distal middle cerebral artery occlusion, the expression of Drp1 and Lysine Acetyltransferase 2A (GCN5L1) was upregulated, and their interaction was promoted, accompanied by increased mitochondrial fission, mtROS accumulation, and cell apoptosis. Furthermore, overexpression of GCN5L1 significantly enhanced Drp1 acetylation and mitochondrial fission, whereas knockdown of GCN5L1 inhibited these processes. Mechanistically, ischemia/hypoxia‐induced Drp1 phosphorylation through cyclin‐dependent kinase 5 (CDK5)‐mediated activation of adenosine 5‘‐monophosphate (AMP)‐activated protein kinase (AMPK), which, in turn, facilitated the interaction between GCN5L1 and Drp1, thereby enhancing Drp1 acetylation and mitochondrial fission. Notably, inhibition of AMPK effectively alleviated Drp1 acetylation and mitochondrial fission, thereby reducing ischemic damage. These findings highlight the crucial role of GCN5L1 in ischemia/hypoxia‐induced mitochondrial fission and provide potential molecular targets for the treatment of ischemic injury [96]. Mitochondrial fission is not only influenced by posttranslational modifications at the protein level, but RNA modifications also play a crucial role in this process. Studies have shown that the fat mass and obesity‐associated protein reduces the m6A modification level of Src family tyrosine kinase (FYN) mRNA, thereby inhibiting FYN expression. This, in turn, leads to a decrease in FYN protein levels, a reduction in the phosphorylation of Drp1 bound to FYN, and ultimately results in diminished mitochondrial fission, suppressed ferroptosis, and enhanced cell viability [97]. In conclusion, both mitophagy and mitochondrial dynamics play pivotal roles in HIBD. In‐depth exploration of their underlying mechanisms provides valuable insights into the pathological processes of injury and identifies potential therapeutic targets. Future research, building upon these findings, is expected to develop more effective intervention strategies, ultimately benefiting patients.

#### 3.1.2 Myocardial Ischemia/Reperfusion

In coronary artery disease, plaques or thrombi can cause rapid occlusion, limiting blood flow to the heart. This may lead to extensive myocardial cell death, preventing the heart from effectively pumping blood to vital organs. Although emergency coronary reperfusion techniques such as coronary artery bypass grafting or percutaneous coronary intervention can limit myocardial cell death, studies have shown that a significant proportion of myocardial cells still undergo cell death during the initial few minutes of reperfusion [98, 99, 100]. This phenomenon is known as myocardial IRI. Similar to HIBD, MQC also plays a critical role in cardiac IRI [101]. MQC primarily involves processes such as mitophagy, fission, and fusion. These processes are interconnected and work synergistically to determine the fate of the heart during IRI. We will explore each of these mechanisms in detail. During IRI, nuclear receptor subfamily 4 group a member 1 (NR4A1) increases mitochondrial fission factor (Mff)‐mediated mitochondrial fission and inhibits FUNDC1‐mediated mitophagy, leading to mitochondrial homeostasis imbalance, impaired clearance of damaged mitochondria, and the activation of pan‐apoptotic programs [102]. Similarly, the researchers also found that upregulated NR4A1 promotes Fis1‐mediated mitochondrial fission and inhibits Parkin‐driven mitophagy, thereby exacerbating cardiac damage [103]. Additionally, checkpoint kinase 1 (CHK1) mitigates oxidative stress, maintains mitochondrial metabolism, and promotes mitochondrial biogenesis and autophagy to restore mitochondrial homeostasis during IRI. CHK1 phosphorylates sirtuin 1 (SIRT1), inhibiting its degradation, thereby maintaining mitochondrial dynamics and cardioprotective effects, while inhibition of SIRT1 abolishes these protective effects [104]. As research on mitophagy in IRI deepens, an increasing number of researchers are focusing on identifying compounds that target this pathway. Through network pharmacology analysis, the study reveals that the Yiqi Huoxue formula exerts a protective effect against myocardial IRI by regulating autophagy and mitigating oxidative stress, with a particular emphasis on the modulation of mitophagy as a key mechanism [105]. Additionally, gastrodin (GAS) activates the PINK1/Parkin pathway to enhance mitophagy, promoting the clearance of damaged mitochondria and offering cardiac protection [106]. Quercetin regulates DNA‐dependent protein kinase catalytic subunit (DNA‐PKcs) stability through SIRT5 desuccinylation, and together they coordinate the “mitophagy‐unfolded protein response,” maintaining mitochondrial membrane and genomic integrity, as well as mitochondrial dynamics and energy metabolism. Notably, quercetin may synergistically regulate mitophagy and the unfolded protein response through the interaction between DNA‐PKcs and SIRT5, thereby improving mitochondrial function and promoting cardioprotective effects (Figure 2B) [107].

To gain a deeper understanding of the role of MQC in IRI, in addition to studying the mechanisms of mitophagy, mitochondrial fission and fusion also play a critical role in maintaining mitochondrial function and cell survival. The study found that Kruppel like factor 4 (Klf4) is significantly downregulated in I/R. Further mechanistic research revealed that Klf4 deficiency exacerbates myocardial IRI by upregulating ROCK1 expression, promoting dephosphorylation of Drp1 at Ser‐616, and enhancing Drp1‐mediated mitochondrial fission. This finding suggests that restoring Klf4 expression could be a potential cardiac protective strategy for alleviating IRI [108]. Additionally, long noncoding RNA (lncRNA) Oip5‐as1 has been extensively studied. Oip5‐as1 is significantly downregulated in IRI. It selectively interacts with A‐kinase anchoring protein 1 and calcineurin (CaN) proteins, inhibiting CaN activation and reducing the dephosphorylation of Drp1 at Ser‐637. This limits Drp1 translocation to the mitochondria and its involvement in mitochondrial fission. This mechanism further elucidates the protective role of Oip5‐as1 in IRI [109]. Therefore, many researchers often explore strategies aimed at promoting mitochondrial fusion when searching for effective therapeutic approaches. Gypenoside XVII (GP‐17) upregulated the downregulated Mfn2 levels in I/R, protecting mitochondrial function by regulating the balance between mitochondrial fusion and fission [110]. Furthermore, researchers have found that cordycepin upregulates the expression of Mfn2 through an AMPK‐dependent signaling pathway, promoting mitochondrial fusion and protecting mitochondrial function [111]. In addition to searching for effective therapeutic agents in natural products, researchers have also targeted mitochondrial fusion proteins using small molecule compounds. For example, a small molecule agonist, S89, specifically targets the endogenous Mfn1, promoting mitochondrial fusion. This compound effectively mitigates mitochondrial damage induced by I/R and protects the mouse heart from IRI [112]. In conclusion, MQC plays a crucial role in myocardial IRI. Strategies that regulate mitochondrial fusion, fission, and autophagy have demonstrated protective potential through natural products and small molecule compounds. However, further exploration is needed to precisely modulate these mechanisms and translate them into clinical applications.

#### 3.1.3 Renal I/R

Renal IRI refers to kidney damage that occurs during the restoration of blood flow following an interruption [113]. This injury is commonly observed in conditions such as embolic or thrombotic events, shock, hypotension, cardiac bypass surgery, organ procurement, and other kidney insults. I/R can lead to a rapid decline in kidney function, increasing the risk of complications, and in severe cases, may result in chronic kidney disease or end‐stage renal disease requiring dialysis [114, 115]. Due to the mismatch between oxygen supply and demand, there is a progressive loss of tubular epithelial cells, impairing the kidney's ability to maintain homeostasis of metabolic waste products, as well as disrupting water and electrolyte balance, leading to a range of associated morbidities [115]. The kidneys, second only to the heart in mitochondrial content, have high energy demands, making them particularly susceptible to cellular damage and tissue dysfunction when mitochondrial function is impaired [108, 109]. Extensive research has shown that mitophagy and changes in mitochondrial dynamics play crucial roles in IRI, directly influencing the progression and recovery of the disease [115, 116, 117, 118, 119]. Therefore, aligning with the discussions on the brain and heart, we will delve deeper into the regulatory roles of mitophagy and mitochondrial dynamics in IRI. Notably, these mechanisms play a central role in preserving mitochondrial function, maintaining cellular homeostasis, and ameliorating disease progression. Their pivotal contributions not only enhance our understanding of the pathophysiological mechanisms underlying IRI but also provide critical theoretical foundations for the development of novel therapeutic strategies. Research has found that the deletion of the C‐terminal transactivation domain (CTAD) of HIF‐1α exacerbates hypoxia‐induced kidney damage. Mechanistic studies show that HIF‐1α CTAD transcriptionally regulates hexokinase 2 (HK2), thereby alleviating tubular injury. Deletion of HK2 exacerbates kidney damage by inhibiting mitophagy, while activation of mitophagy using urolithin A significantly protects HIF‐1α CTAD knockout mice from hypoxia‐induced renal injury. This indicates that HIF‐1α plays a protective role IRI [120]. In contrast, other proteins exert opposing effects during IRI, aggravating kidney damage. For instance, acyl‐CoA synthetase family member 2 (ACSF2) is specifically highly expressed in renal tubular cells and localized in mitochondria. Studies have shown that silencing ACSF2 enhances IRI‐induced mitophagy, promotes mitochondrial function recovery, and significantly reduces mitochondrial superoxide production [121]. Furthermore, 8‐oxoguanine DNA glycosylase (OGG1) is induced during IRI. It has been found that OGG1 negatively regulates mitophagy by inhibiting the PINK1/Parkin pathway, thus exacerbating kidney ischemic injury [122]. Additionally, deletion of pannexin 1 (PANX1) promotes mitophagy and alleviates tubular cell death, oxidative stress, and mitochondrial damage. The specific mechanism is that PANX1 regulates the ATP–P2Y–mTOR signaling pathway, interfering with the mitophagy process, which in turn affects the extent of renal injury (Figure 2C) [123].

In addition to the mechanism of mitophagy, mitochondrial dynamics play a critical role in maintaining mitochondrial function and cell survival. Studying these processes helps deepen the understanding of the role of MQC in IRI [115]. Genetic knockout of STE20‐like kinase 1 (Mst1) significantly improved renal function, alleviated reperfusion‐induced tubular epithelial cell apoptosis, and reduced susceptibility to IRI. Mechanistic studies revealed that Mst1 regulates mitochondrial fission by inducing phosphorylation of Drp1 at Ser‐616 and promoting F‐actin polymerization. More importantly, Mst1 modulates Drp1 posttranslational modifications and F‐actin stability via the GSK3β–p53 signaling pathway. Inhibition of this pathway reduces mitochondrial fission, thereby enhancing cell survival under IRI conditions [124]. Therefore, the use of Drp1 inhibitor P110 in IRI helps maintain mitochondrial homeostasis and reduces renal injury [125]. With the development of research on mesenchymal stromal cell (MSC)‐derived extracellular vesicle (EVs), an increasing number of studies have identified them as a potential therapeutic approach. EVs derived from human Wharton's jelly MSCs ameliorate IRI by inhibiting mitochondrial fission through miR‐30b/c/d [126]. In conclusion, MQC plays a critical role in IRI, directly influencing the progression and recovery of the condition. In‐depth exploration of these mechanisms not only enhances our understanding of the pathophysiology of IRI but also provides theoretical support for the development of novel therapeutic strategies.

### 3.2 Inflammatory Diseases

#### 3.2.1 Sepsis

Sepsis is a systemic inflammatory response syndrome triggered by infection, often accompanied by acute organ dysfunction, with a high mortality rate and long‐term functional impairments in many survivors [127]. Initially referred to as “blood poisoning,” the pathophysiology of sepsis has gradually been recognized as resulting from excessive host immune activation rather than solely from the invading pathogen. In 1992, an international consensus panel defined sepsis as a systemic inflammatory response to infection and introduced the term “severe sepsis” to describe cases complicated by acute organ dysfunction [128]. The pathogenesis of sepsis is closely associated with aberrant immune responses, where excessive activation of immune cells, abnormal cytokine release, and increased vascular permeability can lead to organ damage. Recent studies have highlighted the critical role of mitochondrial dysfunction in the development of sepsis, particularly in maintaining endothelial cell homeostasis, regulating immune cell metabolism, and modulating inflammatory responses. Mitochondria not only serve as the primary energy source for cells but also participate in calcium homeostasis, ROS generation, and the regulation of cell death. Therefore, MQC, as a key mechanism in maintaining mitochondrial function, is essential for preventing organ damage in sepsis [129]. By regulating mitochondrial morphology, function, and quality, MQC can modulate immune and inflammatory responses, offering new therapeutic targets and strategies for the prevention and treatment of sepsis. Acute lung injury (ALI) is a leading cause of mortality in sepsis patients, primarily due to proinflammatory endothelial changes and endothelial permeability defects [130]. Research has shown that mitochondrial dysfunction plays a critical role in the pathogenesis of sepsis‐induced ALI. SIRT1, a histone deacetylase (HDAC), exerts protective effects in sepsis‐induced ALI by regulating inflammation, mitophagy, and cellular homeostasis, thereby reducing excessive inflammatory responses. Mechanistically, the absence of SIRT1 disrupts mitochondrial homeostasis in lung endothelial cells. This disruption triggers excessive production of mtROS and release of mtDNA into the cytoplasm. These changes subsequently activate the NLR family pyrin domain containing 3 (NLRP3) inflammasome and the Stimulator Of Interferon Response CGAMP Interactor 1 (STING) pathway, intensifying the inflammatory response and contributing to organ damage [131]. Additionally, the lack of SIRT3 enhances lung endothelial pyroptosis by disrupting mitophagy, leading to the activation of the NLRP3 inflammasome and worsening sepsis‐induced ALI [132]. Furthermore, studies have shown that inhibiting the methylation of the miR‐138‐5p promoter can alleviate pyroptosis and pulmonary inflammation induced by ALI. In sepsis models, the activation of miR‐138‐5p promoter methylation leads to the downregulation of miR‐138‐5p expression, thereby promoting the activation of the NLRP3 inflammasome and inducing pyroptosis. The process of mitophagy eliminates damaged mitochondria, thereby decreasing cytoplasmic mtDNA levels. This mechanism may also alleviate pulmonary inflammation by reversing the hypermethylation of the miR‐138‐5p promoter [133]. In conclusion, targeting the regulation of mitophagy holds promise as a potential therapeutic strategy to alleviate ALI. Consequently, Thus, investigating whether mitophagy‐targeting compounds can effectively alleviate ALI has emerged as a highly relevant and promising research direction.

Sepsis not only causes lung injury but also leads to multiple organ dysfunction, with cardiac injury being one of the major causes of mortality in sepsis patients. Sepsis‐induced cardiac injury, commonly referred to as SIC, is characterized by impaired myocardial contractility and relaxation [134]. In severe cases, it can progress to heart failure. Therefore, MQC plays a crucial role in SIC. Cardiac‐specific overexpression of Beclin‐1 promotes autophagy and inhibits the mTOR signaling pathway, thereby improving SIC. Mechanistically, Beclin‐1 primarily exerts its mitochondrial protective function through the PINK1–Parkin pathway [135]. In SIC, cardiac PPARα expression is significantly downregulated, and cardiomyocyte‐specific PPARα deficiency exacerbates mitochondrial damage. Mechanistically, PPARα deficiency leads to mitochondrial dysfunction, characterized by reduced ATP production, decreased mitochondrial complex activity, and abnormal expression of Drp1/Mfn1 proteins, which in turn causes disturbances in fatty acid metabolism, exacerbating mitochondrial damage. Ultimately, this promotes mitophagy and mitochondrial‐dependent apoptosis, driving the onset and progression of SIC [136]. In conclusion, MQC plays a central role in sepsis and its associated organ damage. Targeting the regulation of MQC to modulate mitophagy and mitochondrial function may emerge as a crucial therapeutic strategy to alleviate sepsis‐induced injury.

#### 3.2.2 Rheumatoid Arthritis

Rheumatoid arthritis (RA) is a chronic inflammatory joint disease primarily affecting the joints, leading to cartilage and bone damage, which can result in functional impairment. In addition to joint symptoms, RA may also present with extra‐articular manifestations such as rheumatoid nodules, pulmonary involvement, and vasculitis, as well as systemic comorbidities, particularly affecting the vasculature and metabolism [137]. The pathogenesis of RA is complex, with mitochondrial dysfunction playing a significant role in its development [138]. MQC, which regulates mitophagy, dynamics, and function, is critically involved in the inflammatory response and joint destruction associated with RA. As research on MQC progresses, therapeutic strategies targeting this mechanism may offer new treatment options for RA patients, potentially improving prognosis and quality of life. The study found that under hypoxic conditions, fibroblast‐like synoviocytes regulate the expression of BNIP3 and NLRP3 through the transcription factor HIF‐1. Hypoxia‐induced BNIP3‐mediated mitophagy suppresses pyroptosis primarily by reducing intracellular ROS levels [139]. Triggering receptor expressed on myeloid cells 1 (TREM1) is highly expressed in the synovial tissue of arthritis mice and is closely associated with aberrant mitophagy. Mechanistically, overexpression of TREM1 significantly reduces the protein levels of PINK1 and LC3B, leading to impaired mitophagy. Further studies revealed that silencing E2F1 significantly reversed the upregulation of TOMM40, mitophagy damage, and ROS accumulation in TREM1‐overexpressing cells, thereby alleviating the damage [140]. Based on these mechanisms, we will next explore potential therapeutic agents targeting mitophagy and delve into their mechanisms in alleviating arthritis and its associated pathological processes. This provides a theoretical basis for the development of new therapeutic strategies. Researchers developed a two‐dimensional piezoelectric nanosheet Fe/BiOCl with ultrasound catalytic activity, which can efficiently generate electrons under ultrasound stimulation, thereby consuming H^+^ in the mitochondrial outer membrane and disrupting the H^+^ supply in the mitochondrial matrix. This process causes mitochondrial membrane potential depolarization, triggering mitophagy in inflammatory regions and eliminating the source of ROS regeneration, thus alleviating RA [141]. Clinically, Guizhi Shaoyao Zhimu Decoction (GSZG), as a traditional Chinese medicine formula, is commonly used to treat RA. Mechanistically, GSZG exerts its protective effects by inhibiting the generation of osteoclast precursors through the PINK1/Parkin‐mediated mitophagy pathway, thereby suppressing the ROS/nuclear factor kappa‐B (NF‐κB) signaling axis [142].

#### 3.2.3 Multiple Sclerosis

Multiple sclerosis (MS) is an autoimmune disease characterized by inflammatory demyelination and neurodegenerative changes in the central nervous system (CNS), primarily driven by immune attacks mediated by autoreactive T cells [143]. Its hallmark pathological features include inflammatory plaque formation in cerebral and spinal white matter, leading to axonal damage and progressive neurological dysfunction, often culminating in disability with no definitive cure. Notably, mitochondrial dysfunction plays a pivotal role in MS pathogenesis [144]. Patients exhibit widespread mitochondrial respiratory chain defects and transport abnormalities, resulting in disrupted neuronal energy metabolism. These pathological alterations not only amplify immune‐mediated demyelination during acute inflammatory phases but also promote sustained neuronal degeneration in chronic progressive forms (including secondary progressive MS and primary progressive MS) through chronic bioenergetic insufficiency [145]. Mitochondrial dysfunction and neuroinflammation reciprocally exacerbate each other, forming a self‐reinforcing cycle that drives MS progression from transient inflammation to permanent neurological damage. Recent studies have revealed that dysregulation of MQC mechanisms serves as a critical molecular bridge linking bioenergetic failure to neuroinflammation. In demyelination models, FKBP prolyl isomerase 5 (FKBP5) knockout mice exhibit significantly delayed myelin repair compared with wild‐type counterparts. Mechanistic investigations demonstrate that the demyelinated microenvironment activates PPAR‐γ, thereby upregulating FKBP5 expression to enhance PINK1/Parkin‐mediated mitophagy. This pathway promotes the clearance of damaged mitochondria, maintains energy homeostasis, reduces oxidative stress, and ultimately facilitates remyelination [146]. Genetic analyses have further elucidated the link between innate immune regulation and mitophagy. Sequencing of peripheral blood mononuclear cells from 203 MS patients and 1000 healthy controls identified a natural variant (G140E) in the autophagy receptor CALCOCO2/NDP52, which was significantly associated with reduced MS susceptibility [147]. In summary, the dysregulation of MQC mechanisms, through the interplay between bioenergetic collapse and neuroinflammatory cascades, constitutes a central hub driving the pathological progression of MS. Targeting key nodal points within the MQC network may offer novel therapeutic strategies that concurrently achieve immunomodulation and neuroprotection in MS treatment.

### 3.3 Neurodegenerative Diseases

#### 3.3.1 Alzheimer's Disease

Alzheimer's disease (AD) is a chronic neurodegenerative disorder characterized by progressive cognitive decline and neuronal death. Its core pathological hallmarks include extracellular β‐amyloid (Aβ) plaque deposition, intraneuronal neurofibrillary tangles formed by hyperphosphorylated Tau protein, and widespread loss of synapses and neurons [148]. Recent studies further indicate that mitochondrial dysfunction serves as a critical driver of early pathological events in AD, interacting with Aβ and Tau pathologies to collectively exacerbate neurodegenerative damage [149]. MQC is a central mechanism that maintains mitochondrial functional integrity, regulating processes such as mitophagy and dynamic balance, thereby ensuring neuronal energy metabolism and survival. However, in AD, dysregulation of the MQC system leads to disturbances in mitochondrial bioenergetics, accumulation of ROS, and aberrant activation of inflammatory signaling pathways. These changes not only directly impair neuronal function but also may exacerbate the production of Aβ and tau pathology, creating a vicious cycle that further aggravates the onset and progression of AD.

Therefore, restoring mitophagy is considered a potential therapeutic strategy for treating AD [150, 151, 152]. Studies have shown that in AD patients and animal models, the deficiency of the neuron‐specific Bcl‐2 family member BOK is closely associated with mitochondrial damage and impaired autophagic function. BOK regulates Parkin‐mediated mitophagy through competitive binding with the myeloid cell leukemia sequence 1 (MCL1)/Parkin complex, thereby promoting the translocation of Parkin to damaged mitochondria to initiate the autophagic process. Further research revealed that overexpression of BOK can alleviate the mitophagy defect and mitochondrial dysfunction in hippocampal neurons of APP/PS1 mice, leading to improved cognitive function [153]. Given the key role of mitophagy in AD pathology, restoring mitophagy has become a promising therapeutic strategy in recent years. Recent research on natural products has made significant progress, with scientists discovering that Honokiol (HKL, C18H18O2), a compound extracted from the bark of Magnolia officinalis, demonstrates potential anti‐AD effects. In APP/PS1 mice treated with HKL, activation of mitophagy and the mitochondrial unfolded protein response were observed, accompanied by reduced oxidative stress and improved mitochondrial dynamics [154]. In the development of compounds targeting mitophagy, researchers have identified a small molecule, UMI‐77, which can safely and effectively induce mitophagy. UMI‐77 binds to MCL1, enhancing its function as a mitophagy receptor protein, thereby promoting its interaction with LC3A to induce mitophagy. UMI‐77 effectively ameliorates cognitive decline in an AD mouse model [155]. In addition to exogenous compounds, endogenous molecules in the human body also exert protective effects by regulating the mitophagy pathway. Researchers found that MT treatment can reverse these defects by improving the fusion of mitophagosomes with lysosomes, alleviating Aβ pathology, restoring mitochondrial function, and enhancing cognitive function. The study also revealed that concurrent treatment with chloroquine blocked the positive effects of MT on behavioral and biochemical outcomes, suggesting that MT could be a potential therapeutic candidate for AD [156]. In summary, from natural product extracts to synthetically designed small molecules and endogenous regulatory factors, multidimensional interventions targeting mitophagy have demonstrated significant potential in improving the pathological progression of AD. Future research should further elucidate the specificity and synergistic mechanisms of different inducers and optimize dosing strategies through preclinical models to advance these therapies toward precision and personalized treatment.

#### 3.3.2 Parkinson's Disease

Parkinson's disease (PD) is a neurodegenerative disorder characterized by the progressive loss of dopaminergic (DA) neurons in the substantia nigra and the formation of Lewy bodies [157]. Clinically, it manifests with motor dysfunctions such as tremor, rigidity, and bradykinesia, accompanied by nonmotor symptoms including olfactory dysfunction and autonomic dysregulation [158]. Recent studies have shown that the imbalance in MQC mechanisms plays a crucial role in the pathogenesis of PD. Mitochondrial dysfunction, including reduced activity of respiratory chain complexes, accumulation of ROS, and mtDNA mutations, is commonly observed in PD patients and models. Furthermore, disruptions in key MQC processes, such as mitochondrial dynamics imbalance, impaired autophagic clearance, and abnormalities in the PINK1/Parkin pathway, further exacerbate the damage to DA neurons [159]. Investigating the relationship between MQC and PD is of great significance for understanding the disease mechanisms and developing therapeutic strategies.

In PD, mutations in the α‐synuclein (α‐Syn) gene lead to familial PD, while aberrant αSyn is a key pathological hallmark of idiopathic PD. This pathological alteration results in mitochondrial dysfunction, which in turn promotes DA neuronal degeneration. Studies have shown that ubiquitin‐specific peptidase 30 (USP30) knockout mice exhibit protection against behavioral deficits, enhanced mitophagy, reduced S129‐phosphorylated α‐Syn, and alleviated loss of DA neurons in the substantia nigra. These findings were further validated using the selective USP30 inhibitor MTX115325, which demonstrated favorable drug‐like properties [160]. In addition, researchers found that the expression level of circEPS15 was significantly reduced in the plasma of PD patients, and its expression level was negatively correlated with the severity of PD motor symptoms. Further experimental results showed that overexpression of circEPS15 protected DA neurons in vitro and in vivo, alleviating neurodegeneration induced by neurotoxins. Moreover, the study revealed that circEPS15 acted as a sponge for miR24‐3p, stabilizing the expression of its target gene PINK1, thereby enhancing PINK1–PRKN‐dependent mitophagy, removing damaged mitochondria, and maintaining mitochondrial homeostasis [161]. Disruption of lncRNA expression is closely associated with the pathological progression of PD. Studies have shown that a novel lncRNA, “NR_030777,” exerts a significant neuroprotective effect against paraquat‐induced neurodegeneration by regulating the mitophagy pathway. At the molecular level, NR_030777 interacts directly with CDK1 and regulates the phosphorylation modification of DRP1, a key protein involved in mitochondrial fission. Specifically, NR_030777 enhances the phosphorylation level of Drp1 at Ser‐616 (p‐Drp1 Ser‐616), promoting mitochondrial fission, which in turn activates the mitophagy pathway and ultimately protects neurons. Targeting mitophagy has become a key direction in exploring therapeutic strategies for PD. Wang et al. found that a novel small molecule compound, BL‐918, could significantly promote the occurrence of mitophagy by activating the PINK1/Parkin signaling pathway. Further mechanistic studies indicated that the activation of the PINK1/Parkin pathway induced by BL‐918 depends on dynamic changes in mitochondrial membrane potential (ΔΨm) and the regulation of mitochondrial permeability transition (MPT) pore (mPTP). Additionally, research confirmed that BL‐918 could significantly alleviate the progression of PD in a 1‐methyl‐4‐phenyl‐1,2,3,6‐tetrahydropyridine‐induced PD mouse model in a PINK1‐dependent manner, providing important experimental evidence for its potential as a therapeutic drug for PD [162]. In conclusion, the pathogenesis of PD is highly complex, with MQC imbalance considered a key factor. Research targeting mechanisms such as mitophagy has made significant progress. Moving forward, further investigation into these mechanisms and the development of more targeted therapeutic strategies holds promise for improving patient quality of life and delaying disease progression.

## 4 The Role of Mitochondria in Tissue Injury and Repair

Mitochondria, beyond serving as the primary source of ATP through OXPHOS, play multifaceted roles in cellular metabolism, redox regulation, signaling pathways, and the execution of cell death. Their double‐membrane architecture supports efficient energy production and underpins their critical function in maintaining cellular integrity, particularly under conditions of stress or injury. Recent evidence indicates that mitochondrial dysfunction is closely linked to the initiation, progression, and resolution of a broad spectrum of diseases. In addition to regulating intracellular energy homeostasis and redox balance, mitochondria serve as central mediators of programmed cell death. The release of proapoptotic factors such as cytochrome *c* from the intermembrane space activates the caspase cascade, leading to apoptosis. Moreover, mitochondrial impairment can trigger alternative cell death pathways, including necrosis, pyroptosis, and autophagy‐related cell death, thereby amplifying tissue injury. Given these diverse functions, mitochondria are integral not only to disease pathogenesis but also to tissue repair. Elucidating the mechanisms by which mitochondria influence injury response will enhance our understanding of disease biology and may inform the development of targeted interventions. Therapeutic strategies aimed at restoring mitochondrial function, modulating redox homeostasis, or regulating mitophagy represent promising avenues for mitigating tissue damage and promoting regeneration.

### 4.1 Oxidative Stress

#### 4.1.1 Reactive Oxygen Species

ROS have long been regarded as by‐products of mitochondrial respiration, often associated with cellular dysfunction and various diseases [163, 164]. However, recent research has revealed that ROS play far more complex and crucial roles within cells, particularly in relation to mitochondrial function [165]. In the mitochondrial ETC, oxygen acts as the final electron acceptor, driving ATP synthesis while approximately 1–2% of the electrons leak and react with oxygen to produce ROS. While ROS account for only a small fraction (1–2%) of oxygen consumption under normal physiological conditions, their functional importance within the cell is critical [166]. ROS are not merely products of oxidative stress; they are key regulators in processes such as cellular adaptation to environmental changes, gene expression, and cell fate determination. Overall, the interaction between ROS and mitochondria is essential not only for maintaining cellular function and metabolic homeostasis but also for modulating cellular responses in various pathological states [165]. Understanding the complex roles of ROS in cellular physiology and pathology is crucial for developing targeted therapeutic strategies for a wide range of diseases.

In ischemic and hypoxic‐related diseases, ROS, mitochondrial dysfunction, and oxidative stress form a complex and highly interdependent pathological network, which is considered a key driving force behind the onset and progression of cellular damage. ROS can compromise the integrity of the mitochondrial membrane, alter its permeability, and impair energy metabolism, leading to ATP production deficits. These disruptions exacerbate apoptotic and necrotic cell death, creating a vicious cycle that further intensifies tissue damage. In this section, we will primarily focus on the role of ROS in I/R and hypoxia‐related diseases, highlighting their contribution to the pathological processes involved.

In brain ischemia–hypoxia, the abnormal accumulation of ROS triggers a series of cascading reactions that cause significant damage to brain tissue. Neurons in the brain are highly sensitive to energy supply, and under ischemic–hypoxic conditions, the mitochondrial ETC is severely disrupted [167]. This impairs electron transfer, leading to excessive electron leakage and consequently the overproduction of ROS. These excess ROS directly attack mitochondrial membranes, cell membranes, and various biomolecules, such as lipids, proteins, and nucleic acids. Ischemia induces oxidative stress and neuronal ferroptosis. TP53‐induced glycolysis regulatory phosphatase (TIGAR) mitigates brain injury by inhibiting glycolysis, enhancing the pentose phosphate pathway (PPP), generating NADPH and GSH, and scavenging ROS. Prolonged ischemia impairs the PPP, but TIGAR still reduces ferroptosis and alleviates brain damage. The study reveals that ROS are primarily generated by mitochondrial succinate dehydrogenase (SDH) via reverse electron transfer. TIGAR suppresses SDH activity, likely through its mitochondrial translocation and interaction with SDH subunit A, mediating posttranslational modifications (acetylation and succinylation) of SDH. Notably, TIGAR‐mediated SDH inhibition and ferroptosis reduction are independent of the PPP–NADPH–GPX4 pathway, highlighting its distinct antioxidant mechanism [168]. However, in addition to ferroptosis, pyroptosis is also a form of cell death that plays a critical role in ischemic brain injury. The researchers found that overexpression of SIRT3 alleviates cerebral infarction and brain atrophy in HI rats. SIRT3 reduces neuroinflammation by inhibiting microglial activation, decreasing interleukin (IL)‐1β and MPO‐positive cell numbers. Further studies showed that SIRT3 suppresses NLRP3 inflammasome activation, leading to reduced release of proinflammatory cytokines. SIRT3 also improves mitochondrial function, decreases ROS production, and mitigates mitochondrial depolarization. Ultimately, SIRT3 effectively reduces neuronal apoptosis, providing neuroprotective effects [169]. Moreover, the researchers found that uncoupling protein 2 (UCP2) also plays a significant role in pyroptosis induced by cerebral IRI. In UCP2‐deficient mice, tissue pathological changes and cell apoptosis after cerebral IRI were markedly exacerbated. UCP2 deficiency also enhanced the activation of NLRP3 inflammasomes in neurons, especially under hyperglycemic conditions. In vitro, UCP2 knockout further promoted NLRP3 inflammasome activation and ROS production in HT22 cells. In conclusion, UCP2 deficiency plays a critical role in exacerbating cerebral IRI under hyperglycemia and may serve as a potential therapeutic target [170]. In recent years, a wealth of research has underscored the importance of diverse intervention approaches. These strategies play a crucial role in modulating the generation of ROS and alleviating the harm caused by brain ischemia–hypoxia injury. Clinically, raloxifene is primarily used for the prevention and treatment of osteoporosis in postmenopausal women. However, recent studies have revealed its significant neuroprotective effects in OGD/reoxygenation models. Raloxifene enhances cell survival, preserves mitochondrial membrane potential, and reduces lipid peroxidation and ROS production. These findings suggest that raloxifene directly improves mitochondrial function, highlighting its potential therapeutic role in hypoxic–ischemic brain injury [171]. Danshen injection is commonly used in clinical practice for the treatment of cardiovascular diseases such as coronary heart disease and angina pectoris, which are caused by blood stasis, and it exerts effects of promoting blood circulation and unblocking meridians. Studies have shown that in a middle cerebral artery occlusion model, Danshen injection not only improves neurological function scores and reduces infarction rates but also inhibits inflammation and oxidative stress, thereby alleviating tissue damage. The underlying mechanism may involve the suppression of the HIF‐1α/CXCR4/NF‐κB signaling pathway, and overexpression of HIF‐1α leads to the loss of Danshen's anti‐inflammatory effects, suggesting that it may directly target HIF‐1α [172]. Silymarin is clinically used to treat liver toxicity. Recent studies have shown that silymarin significantly enhances cell viability, reduces apoptosis, and markedly decreases LDH release and ROS generation in neuroblastoma N2a cells. Its neuroprotective effect is dependent on the growth arrest‐specific protein 6 (GAS6) signaling pathway, as silencing GAS6 expression significantly inhibits the neuroprotective effects of silymarin. These findings suggest that GAS6 plays a crucial role in the neuroprotective mechanism of silymarin, highlighting its potential therapeutic value in ischemic stroke and other neurodegenerative conditions [173]. These findings suggest that traditional drugs have new applications in the treatment of ischemic stroke and other neurodegenerative diseases, demonstrating their significant potential as novel neuroprotective agents.

Similarly, strategies that modulate ROS levels to alleviate tissue damage are equally applicable to myocardial IRI. Among these, ginsenoside Rb1 has been shown to effectively reduce infarct size and protect cardiac function by decreasing mitochondrial ROS levels and stabilizing the activity of mitochondrial complex I. Its mechanism of action is closely associated with the regulation of NADH dehydrogenase subunit expression [174]. Crocin exerts cardioprotective effects by suppressing ROS generation and regulating the NLRP3 inflammasome and caspase‐1, significantly reducing the expression of proinflammatory cytokines such as IL‐6, tumor necrosis factor (TNF)‐α, and IL‐1β. Similarly, pNaKtide alleviates IRI by reducing ROS accumulation, demonstrating potential for antioxidant therapy [175]. Similarly, pNaKtide alleviates IRI by reducing ROS accumulation, demonstrating potential for antioxidant therapy [176]. In recent years, the role of materials science in protecting against myocardial IRI has garnered increasing attention. Among these advancements, the administration of exogenous nitric oxide (NO) has been recognized as an effective strategy for treating myocardial infarction (MI) due to its diverse physiological functions [177, 178, 179, 180]. However, during I/R, the restoration of blood flow is often accompanied by excessive production of toxic ROS, which not only exacerbates tissue damage but also diminishes the efficacy of NO‐based therapies. To address this challenge, researchers have developed a novel dual‐functional hydrogel, CS‐B‐NO, synthesized from chitosan modified with boronate ester‐protected dinitrobenzene. This hydrogel releases NO in response to ROS stimulation, effectively restoring the ROS/NO balance following IRI. In a mouse model of myocardial IRI, CS‐B‐NO significantly reduced myocardial damage and adverse cardiac remodeling, enhanced cardiac repair, and improved cardiac function compared with hydrogels that solely release NO. Mechanistically, CS‐B‐NO modulates the ROS/NO balance by activating the antioxidant defense system via the Nrf2–Keap1 pathway, thereby mitigating oxidative stress caused by IRI. Additionally, CS‐B‐NO suppresses NF‐κB signaling to reduce inflammation [181]. In summary, this dual‐functional hydrogel shows significant potential in myocardial protection and functional recovery, making it a promising tool for tissue preservation after IRI. Furthermore, these findings highlight the central role of ROS in IRI and validate the effectiveness of antioxidant and anti‐inflammatory strategies. Notably, the integration of traditional pharmacological approaches with materials science innovations offers a valuable avenue for advancing cardiovascular disease prevention and treatment.

### 4.2 Cell Death

Various forms of cell death are an inevitable outcome in maintaining cellular health and dynamic equilibrium in all living organisms [6, 182]. Programmed cell death, as the primary mechanism of cellular turnover, is tightly regulated by numerous genes. Rigorous MQC is essential for the proper functioning of mitochondria and is closely linked to physiological programmed cell death.

#### 4.2.1 Apoptosis

**FIGURE 3:**
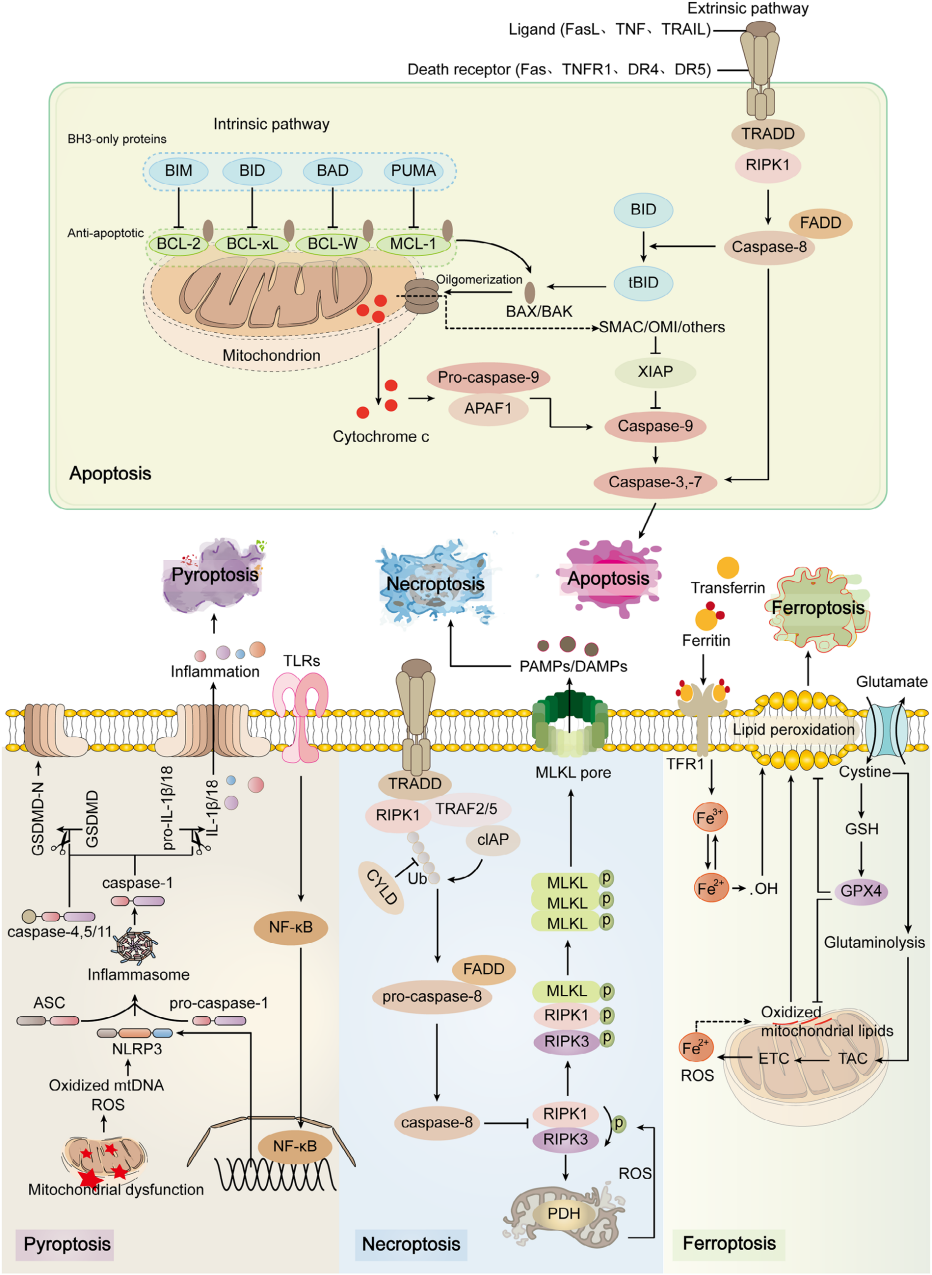
Molecular pathways of cell death. *Apoptosis*. Apoptosis involves two pathways: the intrinsic mitochondrial pathway and the extrinsic death receptor pathway. The intrinsic pathway is triggered by stress signals that activate BAX/BAK, causing MOMP and cytochrome *c* release, which forms the apoptosome to activate caspase‐9 and downstream caspases. The extrinsic pathway starts with death receptor activation, forming the DISC and activating caspase‐8, which cleaves caspase‐3/7. Caspase‐8 also cleaves BID to tBID, linking both pathways and amplifying apoptosis. *Pyroptosis*: In mice, PAMPs are recognized by PRRs, leading to activation of caspase‐1 and caspase‐11, whereas in humans, this pathway is mediated by caspase‐1, ‐4, and ‐5. Activated caspases cleave GSDMD, releasing the GSDMD‐N, which inserts into the plasma membrane to form pores, causing membrane rupture and release of proinflammatory cytokines IL‐1β and IL‐18. MOMP induces the release of mtDNA and ROS; mtDNA acts to activate the NLRP3 inflammasome, while ROS promote inflammation, establishing a positive feedback loop that exacerbates pyroptosis. *Necroptosis*: TNF binding induces TNFR1 trimerization and formation of Complex I, which recruits TRADD, RIPK1, and other factors to maintain cellular homeostasis. Upon dysregulation, activated RIPK1 assembles Complex IIa, composed of FADD and caspase‐8, to induce apoptosis. When caspase‐8 is inhibited, RIPK1 interacts with RIPK3 to form Complex IIb, leading to RIPK3 activation and phosphorylation of MLKL. Phosphorylated MLKL oligomerizes and translocates to the plasma membrane, disrupting membrane integrity and executing necroptosis. *Ferroptosis*: xCT, encoded by *SLC7A11*, mediates the exchange of extracellular cystine and intracellular glutamate. Once inside the cell, cystine is reduced to cysteine, which is essential for GSH synthesis. GSH acts as a cofactor for GPX4, an enzyme that prevents lipid peroxidation. Cysteine deficiency impairs GPX4 activity, enhances TCA cycle flux, and increases ROS production. Fe^2^⁺ drives the Fenton reaction, generating hydroxyl radicals that oxidize polyunsaturated fatty acids, leading to lipid peroxidation. The breakdown of ferritin and release of mitochondrial iron further intensify oxidative stress. Because mitochondrial membranes are close to ROS and iron sources, they are more prone to structural damage. The combination of increased membrane permeability and ROS accumulation promotes ferroptosis. *Abbreviations*: MOMP, mitochondrial outer membrane permeabilization; APAF‐1, apoptosis protease‐activating factor‐1; FADD, Fas‐associating protein with a novel death domain; BCL‐2, B‐cell lymphoma‐2; BIM, Bcl‐2 interacting mediator of cell death; BID, BH3 interacting domain death agonist; BAD, BCL2‐associated agonist of cell death; PUMA, p53 upregulated modulator of apoptosis; BCL‐xL, B‐cell lymphoma‐extra large; MCL1, myeloid cell leukemia‐1; SMAC, second mitochondria‐derived activator of caspases; OMI, high temperature requirement factor A2; XIAP, X‐linked inhibitor of apoptosis protein; Fas, factor‐related apoptosis; TNFR1, TNF receptor type 1; DR4, death receptor 4; DR5, death receptor 5; FasL, Fas ligand; TNF, tumor necrosis factor DED, death effector domain DISC, death‐inducing signaling complex; PAMPs, pathogen‐associated molecular patterns; PRRs, pattern recognition receptors; GSDMD, gasdermin D; DAMP, damage‐associated molecular pattern; NLRP3, NOD‐like receptor thermal protein domain‐associated protein 3; MLKL, mixed lineage kinase domain‐like protein; CYLD, cylindromatosis; SPATA2, spermatogenesis associated 2; SLC7A11, solute carrier family 7 member 11; SLC3A2, solute carrier family 3 member 2; GSH, glutathione; GPX4, glutathione peroxidase 4; TCA, tricarboxylic acid.

Apoptosis is a highly programmed form of cell death that involves a complex array of endogenous and exogenous signaling pathways, ultimately leading to cellular dismantling via caspase cascade activation. It primarily includes intrinsic apoptosis, activated through the mitochondrial pathway, and extrinsic apoptosis, initiated via death receptor signaling. Intrinsic apoptosis relies on mitochondrial rupture and the release of cytochrome *c*, with key regulatory proteins such as BAX and BAK playing pivotal roles in this process (Figure 3) [182]. Extrinsic apoptosis is triggered by death receptors, such as Fas and TNF receptors, which activate caspase cascades to drive cell death. In recent years, MQC has gained significant attention as a critical mechanism for maintaining cellular homeostasis [6]. Mitochondrial dynamics, particularly fission and fusion processes, play a crucial role in regulating apoptosis and maintaining mitochondrial health. It has been revealed that mitochondria are not only central executors of intrinsic apoptosis, but their quality control mechanisms—such as the cross‐regulation between autophagy and apoptosis—are essential in determining cell fate. Therefore, exploring how MQC functions in apoptosis provides valuable insights into the mechanisms of cell death and offers potential therapeutic targets for related diseases. *Intrinsic apoptosis*: Intrinsic apoptosis (also known as the mitochondrial pathway of apoptosis) is a programmed cell death process activated by internal cellular stressors, such as DNA damage, oxidative stress, nutrient deprivation, or mitochondrial dysfunction [183]. This process is primarily regulated by the Bcl‐2 protein family, which controls the balance between proapoptotic and antiapoptotic proteins and modulates mitochondrial function, ultimately determining the cell's fate [182, 184]. *Regulatory role of the Bcl‐2 protein family*: The Bcl‐2 protein family plays a central role in intrinsic apoptosis. This family consists of three categories of proteins: antiapoptotic proteins (e.g., Bcl‐2, Bcl‐xL), proapoptotic effector proteins (e.g., Bax, Bak), and proapoptotic BH3‐only proteins (e.g., Bim, Bid, BAD) [5, 185]. In healthy cells, antiapoptotic proteins inhibit the activation of proapoptotic proteins by binding to them, thus preventing mitochondrial outer membrane permeabilization (MOMP) [186, 187]. However, under conditions of cellular stress, proapoptotic proteins such as Bax and Bak are activated, leading to MOMP and ultimately resulting in cell death. *Role of mitochondrial outer MOMP*: MOMP is a decisive step in intrinsic apoptosis, typically accompanied by the release of cytochrome *c* from the mitochondria. Cytochrome *c* binds to APAF1 to form the apoptosome, which in turn activates caspase‐9, leading to the activation of caspases‐3 and ‐7 [188]. [189] This cascade of events culminates in cellular death. Additionally, MOMP triggers the release of molecules such as SMAC and OMI, which inhibit the antiapoptotic factor X‐linked inhibitor of apoptosis protein (XIAP), thereby further accelerating the apoptotic process. In summary, intrinsic apoptosis is a tightly regulated process orchestrated by the Bcl‐2 protein family, with mitochondrial‐related events at its core, serving as an important programmed cell death mechanism in response to internal stress. In contrast, extrinsic apoptosis is another key apoptotic pathway activated through death receptors on the cell surface. *Extrinsic apoptosis*: Extrinsic apoptosis, also known as death receptor‐mediated apoptosis, is a form of programmed cell death initiated when death receptors on the cell membrane bind to external ligands [190]. This process activates intrinsic apoptotic pathways and ultimately leads the cell toward death. Common death receptors include Fas, TNFR1, and TNF receptor superfamily member 10b (TRAIL‐R2/DR5), which, upon binding with their respective ligands (such as FasL, TNF‐α, TRAIL), trigger complex intracellular signaling events that ultimately determine the cell's fate [182]. A key step in extrinsic apoptosis is the activation of these receptors. For instance, upon TNF‐α binding to TNFR1, the activated TNFR1 recruits adaptor proteins containing death domains, such as TNFRSF1A associated via death domain (TRADD) and receptor interacting serine/threonine kinase 1 (RIPK1), forming complex I. This complex undergoes various ubiquitination modifications (including K63, K48, and K11‐linked polyubiquitination), which regulate downstream signaling molecules and activate prosurvival pathways like NF‐κB, promoting cell survival and inflammatory responses [191, 192, 193, 194]. Additionally, complex I recruits the linear ubiquitin chain assembly complex (LUBAC) complex, which catalyzes M1‐linked ubiquitination, further modulating complex assembly and signaling. However, under certain conditions, such as the absence of cIAP1, cIAP2, or LUBAC, or when RIPK1 ubiquitination is dysregulated, RIPK1 kinase activity may become aberrantly elevated, leading to excessive activation of apoptotic pathways. In this scenario, activated RIPK1 forms complex IIa with FADD and caspase‐8, triggering the caspase cascade and inducing typical apoptotic morphological changes, such as cell shrinkage, chromatin condensation, and DNA fragmentation [195, 196, 197, 198, 199]. Although the initiation mechanisms of extrinsic apoptosis and intrinsic apoptosis are different, they do not exist in isolation. Instead, they are interrelated and act synergistically within the complex network of apoptosis. Extrinsic apoptosis mainly activates a series of signaling cascades through the death receptor pathway, while intrinsic apoptosis centers on the changes in mitochondrial function. The two have interactions at many key nodes.

The classical intrinsic apoptotic pathway involves changes in the MOMP, leading to the release of proapoptotic factors such as cytochrome *c* into the cytoplasm. These released factors subsequently activate caspases, initiating the apoptotic process. Mitochondria also play a key role in oxidative stress responses, where the generation of ROS can lead to mitochondrial damage, thereby promoting cell apoptosis [200, 201]. Research has shown that activation of Yes‐associated protein (YAP) and sarco/ER calcium ATPase 2a (SERCA2a) protects myocardial cells from damage induced by cardiac IRI, including ER stress [202, 203]. This pathway plays a critical role in maintaining mitochondrial redox balance, restoring energy production, reducing calcium overload, inhibiting ER stress, and preventing caspase activation. By facilitating communication between mitochondria and the ER, the YAP/SERCA2a pathway provides a robust protective mechanism against cardiac IRI. NADH:ubiquinone oxidoreductase subunit A13 (NDUFA13) is a novel accessory subunit of mitochondrial complex I, located near subunits with low electrochemical potential. Downregulation of NDUFA13 has been linked to enhanced antiapoptotic properties in tumor cells. Researchers found that NDUFA13 knockout mice (cHet) exhibited normal cardiac function under baseline conditions, but displayed stronger resistance to apoptosis following IRI. cHet mice maintained oxygen consumption through complexes I and II, with elevated cytosolic H_2_O_2_ levels and lower mitochondrial H_2_O_2_ levels. The increased cytosolic H_2_O_2_ promoted STAT3 dimerization, activating antiapoptotic signaling, inhibiting superoxide bursts, and reducing infarct size [204]. In the I/R heart, CaMKII (Ca^2+^/calmodulin‐dependent protein kinase II) activity significantly increases during the early reperfusion period, manifested by the phosphorylation at the Threonine‐17 site. Researchers found that using the CaMKII inhibitor KN‐93 (KN) significantly improved contractile recovery at the end of cardiac reperfusion, reduced the infarct size, decreased the release of lactate dehydrogenase from necrotic cells, reduced apoptosis (TUNEL‐positive cells and caspase‐3 activity), and increased the Bcl‐2/Bax ratio. In isolated cardiomyocytes, KN and CaMKII inhibitory peptide (AIP) were able to prevent spontaneous contractile activity and cell death induced by simulating I/R. Inhibiting the reverse mode Na^+^/Ca^2+^ exchanger (NCX), sarcoplasmic reticulum (SR) function or SR Ca^2+^ release produced similar effects. Conversely, overexpression of CaMKII reduced cell survival rates. Taken together, this study provides the first clear evidence of the crucial role of CaMKII in irreversible IRI. It promotes cell apoptosis and necrosis by integrating the actions of NCX, SR, and mitochondria. This finding contributes to a deeper understanding of the molecular mechanisms involved in IRI [205]. A study found that miR‐762 is a microRNA primarily located in mitochondria and is significantly upregulated after hypoxia/reoxygenation treatment. By knocking out endogenous miR‐762, researchers observed that myocardial cells exhibited reduced ATP levels, increased ROS levels, decreased mitochondrial complex I enzyme activity, and reduced apoptotic cell death under A/R (anoxia/reoxygenation) treatment. Furthermore, miR‐762 knockout improved myocardial IRI in mice. The research revealed that miR‐762 participates in regulating mitochondrial function and myocardial cell apoptosis by modulating the mitochondrial complex I core assembly subunit ND2. This discovery provides new insights into potential therapeutic targets for MI [206].

#### 4.2.2 Necroptosis

**FIGURE 4:**
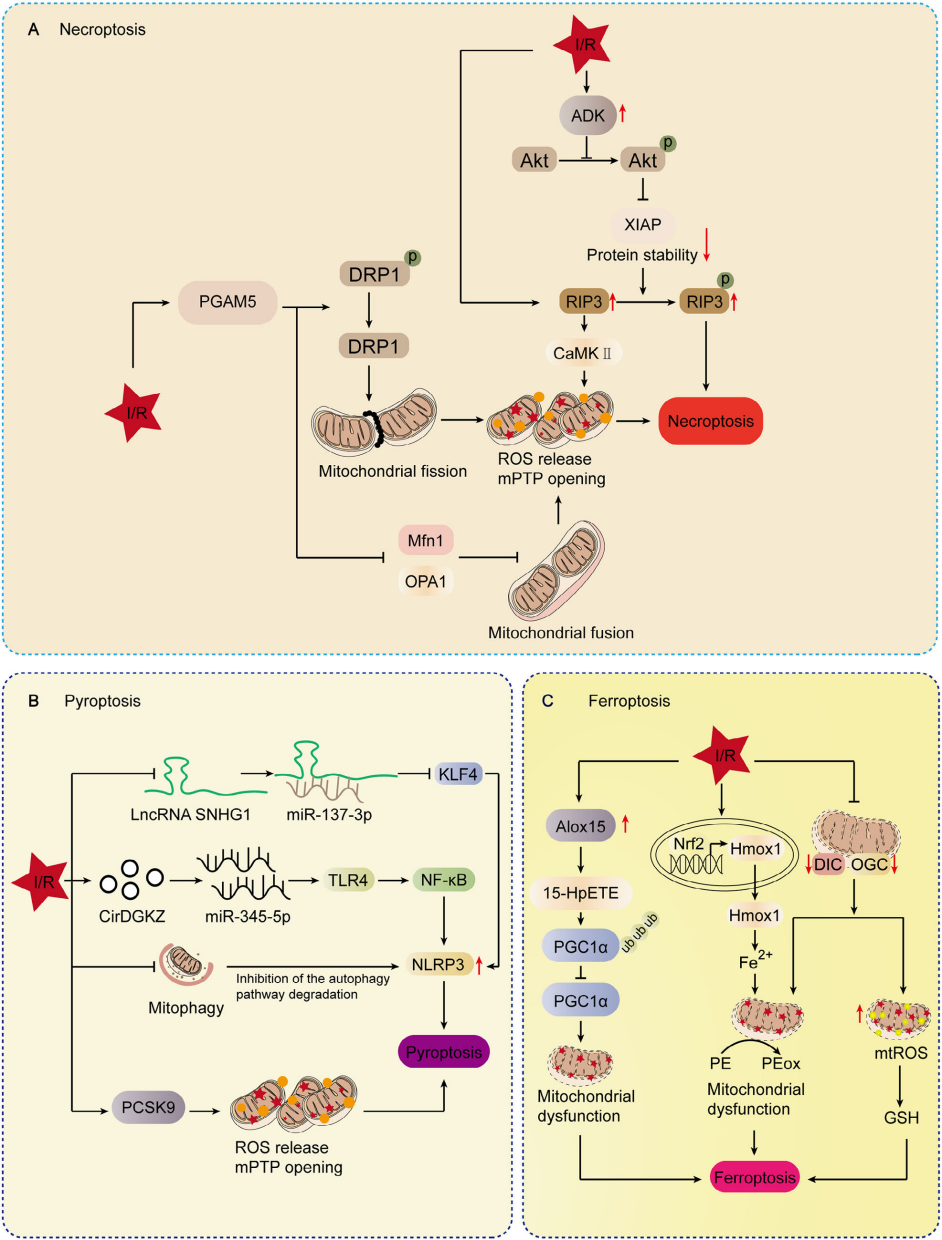
The role of mitochondria in the process of cell death induced by cardiac I/R. (A) PGAM5 is upregulated at both the transcriptional and expression levels during I/R. Deficiency of PGAM5 increases mitochondrial DNA copy number and transcription levels, normalizes mitochondrial respiration, inhibits mitochondrial ROS production, and prevents abnormal mPTP opening during I/R. PGAM5 deficiency disrupts I/R‐induced Drp1 dephosphorylation, partially suppressing mitochondrial fission and ultimately rescuing cardiomyocyte necrosis. Inhibiting ADK can reduce cardiomyocyte necrosis. ADK inhibition decreases MLKL and its phosphorylation, as well as the phosphorylation of CaMKII. XIAP, which is phosphorylated and stabilized through the adenosine receptors A2B and A1/Akt pathways, plays a crucial role in the effects of ADK inhibition on necrosis. RIP3‐induced CaMKII activation may trigger the opening of the mPTP through phosphorylation, oxidation, or both, leading to myocardial necrosis. (B) lncRNA SNHG1 alleviates cardiomyocyte pyroptosis during myocardial ischemia–reperfusion injury by acting as a “sponge” for miR‐137‐3p, thereby regulating the KLF4/TRPV1/AKT axis. Circ‐DGKZ regulates miR‐345‐5p and, through direct interaction, modulates TLR4 in cardiomyocytes. This, in turn, disrupts cardiomyocyte pyroptosis and induces autophagy via the TLR4/NF‐κB axis. PCSK9 can induce mtDNA damage and activate the NLRP3 inflammasome signaling pathway. (C) The Alox15‐derived intermediate metabolite 15‐HpETE promotes the binding of PGC‐1α to the ubiquitin ligase ring finger protein 34, leading to its ubiquitin‐dependent degradation, which causes impaired mitochondrial biogenesis and abnormal mitochondrial morphology, ultimately resulting in ferroptosis. In I/R, Nrf2‐mediated upregulation of Hmox1 leads to systemic accumulation of nonheme iron and induces ferroptosis. DIC and OGC play roles in GSH transport, and their inhibition exacerbates ferroptosis. *Abbreviations*: PGAM5, phosphoglycerate mutase family member 5; ROS, reactive oxygen species; ADK, adenosine kinase; MLKL, mixed lineage kinase domain‐like protein; XIAP, X‐linked inhibitor of apoptosis protein; RIP3, receptor‐interacting protein kinase 3; CaMKII, calcium/calmodulin‐dependent protein kinase II; TRPV1, transient receptor potential vanilloid 1; PCSK9, proprotein convertase subtilisin/kexin type 9; NLRP3, NOD‐like receptor protein 3; Alox15, arachidonate 15‐lipoxygenase; PGC‐1α, peroxisome proliferator‐activated receptor gamma coactivator 1‐alpha; Nrf2, nuclear factor erythroid 2‐related factor 2; Hmox1, heme oxygenase 1; DIC, dicarboxylate carrier; OGC, oxoglutarate carrier; GSH, glutathione.

Necroptosis is a regulated form of cell death that is typically activated when the conventional apoptotic pathway is inhibited, triggered by external signals (e.g., death receptor–ligand binding) or internal signals (e.g., pathogen‐derived nucleic acids) [202]. Unlike apoptosis, necroptosis is characterized by mitochondrial and organelle swelling, plasma membrane rupture, and cellular disintegration, and does not rely on caspase activation (Figure 3). It often serves as an alternative cell death mechanism in response to infection, oxidative stress, or ischemia. In addition to direct cell rupture, necroptosis induces a significant immune response, marked by the infiltration and activation of inflammatory cells. While it plays a protective role in certain contexts, necroptosis can exacerbate tissue damage in pathological conditions, including cardiovascular diseases, infections, and tumors. However, recent studies have shown that mitochondria may also play a regulatory role in certain forms of necroptosis [207, 208]. For instance, necroptosis relies on the production of mitochondrial ROS and the release of mtDNA, which can activate necrosis‐related molecules such as RIPK1/3 and Mixed lineage kinase domain‐like protein (MLKL). Studies have found that gene knockout of phosphoglycerate mutase 5 (PGAM5) can inhibit necrosis in myocardial cells associated with I/R but has no impact on apoptosis. PGAM5 deficiency can normalize mitochondrial respiration, reduce harmful mitochondrial ROS production, and prevent abnormal mitochondrial opening, all of which are associated with improved cardiac function. Furthermore, PGAM5 influences specific molecular events, such as the dephosphorylation of DrpS637, but does not affect others like Drp1 Ser‐616 phosphorylation, partially inhibiting mitochondrial fission and associated protein levels. In summary, PGAM5 plays a crucial role in myocardial cell necrosis by influencing MQC [209]. Research has found that adenosine kinase (ADK), by increasing intracellular and extracellular adenosine levels, reduces infarct size, improves cardiac function, and decreases apoptosis and necrosis in a mouse model. In vitro experiments also showed that ADK inhibition prevented apoptosis and necroptosis in cardiomyocytes under hypoxia/reoxygenation treatment. These effects were associated with the reduction of the activity of key apoptosis and necrosis‐related proteins due to ADK inhibition. Furthermore, ADK's effects were linked to the phosphorylation and stabilization of the XIAP through adenosine receptors A2B and A1/Akt pathways. This study underscores the significant role of ADK in myocardial IRI (Figure 4) [210].

In summary, mitochondria play a crucial regulatory role in necroptosis, particularly in the context of myocardial IRI. By modulating intracellular ROS production, mitochondrial function, and cell death pathways, mitochondria can exert protective effects but may also exacerbate tissue damage in pathological conditions. Therefore, a deeper understanding of mitochondrial involvement in necroptosis could provide new avenues for the treatment of cardiovascular diseases and other related conditions.

#### 4.2.3 Pyroptosis

Pyroptosis is a controlled cell death pathway typically involving members of the gasdermin (GSDMD) protein family, which can lead to the formation of plasma membrane pores [211, 212, 213, 214]. Recent studies have shown that during the early stages of pyroptosis, GSDMD‐NT translocates to the mitochondria, causing mitochondrial depolarization and alterations in membrane permeability, thereby inducing mitochondrial dysfunction. Notably, this process occurs prior to cell death and does not rely on apoptotic proteins such as BCL‐2‐associated X protein (BAX) and bcl‐2 homologous antagonist (BAK). GSDMD‐NT facilitates the translocation of cardiolipin from the inner mitochondrial membrane to the outer membrane, resulting in mitochondrial damage. Additionally, it promotes the release of the polyribonucleotide nucleotidyl transferase 1, which exacerbates mRNA degradation and amplifies the inflammatory response. Therefore, the role of mitochondria in pyroptosis is becoming increasingly evident, suggesting that they may play a crucial regulatory role in this cell death process (Figure 3) [215].

During I/R, the expression of canonical inflammasome components, such as apoptosis‐associated speck like protein containing CARD (ASC) and NLRP3, is increased. At the same time, caspase‐1 and GSDMD are cleaved, leading to the secretion of proinflammatory cytokines IL‐1β and IL‐18 [216, 217, 218, 219]. In fact, during I/R, mitochondrial dysfunction is primarily characterized by inhibition of the ETC, reduction in membrane potential, and decreased ATP synthesis, leading to disturbances in cellular energy metabolism. This results in the disruption of calcium homeostasis, increased oxidative stress, and subsequent damage to mtDNA. Mitochondrial oxidative stress and mtDNA damage are considered key factors in the activation of the NLRP3 inflammasome [220, 221]. ROS also support the mitochondrial recruitment of NLRP3 and ASC, thereby enhancing NLRP3 activation [222, 223]. ROS generated by voltage‐dependent anion channel 1 and the activation of BAX/BAK can also trigger NLRP3 activation. Additionally, calcium overload and its entry into mitochondria may promote the opening of the mPTP, leading to ROS production. This, in turn, facilitates the deubiquitination of NLRP3, further activating the inflammasome and promoting the release of mtDNA, which amplifies NLRP3 activation [224]. In addition, Mitochondrial lipid cardiolipin can directly bind to NLRP3, and interference with its synthesis specifically inhibits the activation of the NLRP3 inflammasome, highlighting the critical role of mitochondria in the activation of the NLRP3 inflammasome [225]. In the upstream mechanisms, proprotein convertase subtilisin/kexin type 9 PCSK9 induces mtDNA damage, activates the NLRP3 inflammasome pathway, and triggers caspase‐1‐dependent pyroptosis. The infarct border zone shows high expression of PCSK9 and GSDMD‐NT. PCSK9 knockout reduces NLRP3 activation, GSDMD‐NT expression, and LDH release. In chronic myocardial ischemia patients, serum PCSK9, NLRP3 signaling, and pyroptosis markers are elevated. These findings suggest PCSK9 regulates pyroptosis via mtDNA damage, highlighting potential targets for treating PCSK9‐related cardiovascular diseases (Figure 4) [226].

#### 4.2.4 Ferroptosis

Ferroptosis is a specific form of necrosis associated with iron‐dependent lipid peroxidation. In the process of ferroptosis, mitochondria may be involved through the generation of ROS and other mechanisms (Figure 3) [227, 228, 229]. In I/R, cardiomyocytes undergo early apoptosis and necrosis, followed by iron accumulation during reperfusion. Accumulation of 15‐lipoxygenase (Alox15) metabolites is notably observed in iron‐induced apoptotic cardiomyocytes. Increased specific expression of Alox15 in the damaged myocardial area colocalizes with cardiomyocytes. Moreover, cardiomyocyte‐specific deletion of Alox15 effectively attenuated IRI in mice and facilitated cardiac function recovery. Further investigations revealed that 15‐hydroperoxy eicosatetraenoic acid (15‐HpETE) promotes the ubiquitin‐dependent degradation of PGC1‐α by facilitating its binding with E3 ubiquitin ligase Parkin, thereby impairing mitochondrial biogenesis and inducing abnormal mitochondrial morphology. Finally, the authors confirmed that ML351, an Alox15‐specific inhibitor, enhances Pgc1α protein levels, suppresses iron accumulation in cardiomyocytes, protects damaged myocardium, and promotes cardiac function recovery [230]. Research has found that typical features of ferroptotic cell death in cardiomyocytes treated with DOX are observed, even in mice with defects in apoptosis‐ and necrosis‐related genes. Further investigation revealed that DOX induces a significant upregulation of heme oxygenase‐1 (Hmox1) in the heart, leading to iron accumulation and subsequently inducing ferroptosis. This process is triggered by Nrf2‐mediated upregulation of Hmox1, with the release of free iron being a key factor in inducing cardiac damage. Additionally, the study also found that ferroptosis is primarily caused by the accumulation of free iron within mitochondria and lipid peroxidation, rather than necrosis driven by MPT. Therefore, targeting both ferroptosis and MPT‐driven necrosis simultaneously can effectively reduce the size of the infarct area, thereby improving cardiac remodeling and offering new strategies for the treatment of IRI. This research highlights mitochondrial mechanisms and provides promising strategies for the prevention of myocardial diseases (Figure 4) [231].

Mitochondria play a central role in cell death pathways, with their functions regulated by various endogenous and exogenous factors. Studies have identified oxidative stress, calpain‐1 activation, and the translocation of apoptosis‐inducing factor from mitochondria to the nucleus as critical mechanisms driving cardiomyocyte death, particularly in the context of myocardial IRI [232]. Elucidating the specific roles and regulatory mechanisms of mitochondria in these processes is essential for understanding the molecular basis of cell death and developing therapeutic strategies. This remains a highly active area of research, with ongoing efforts to uncover further details and advance potential treatments.

### 4.3 Inflammation

**FIGURE 5:**
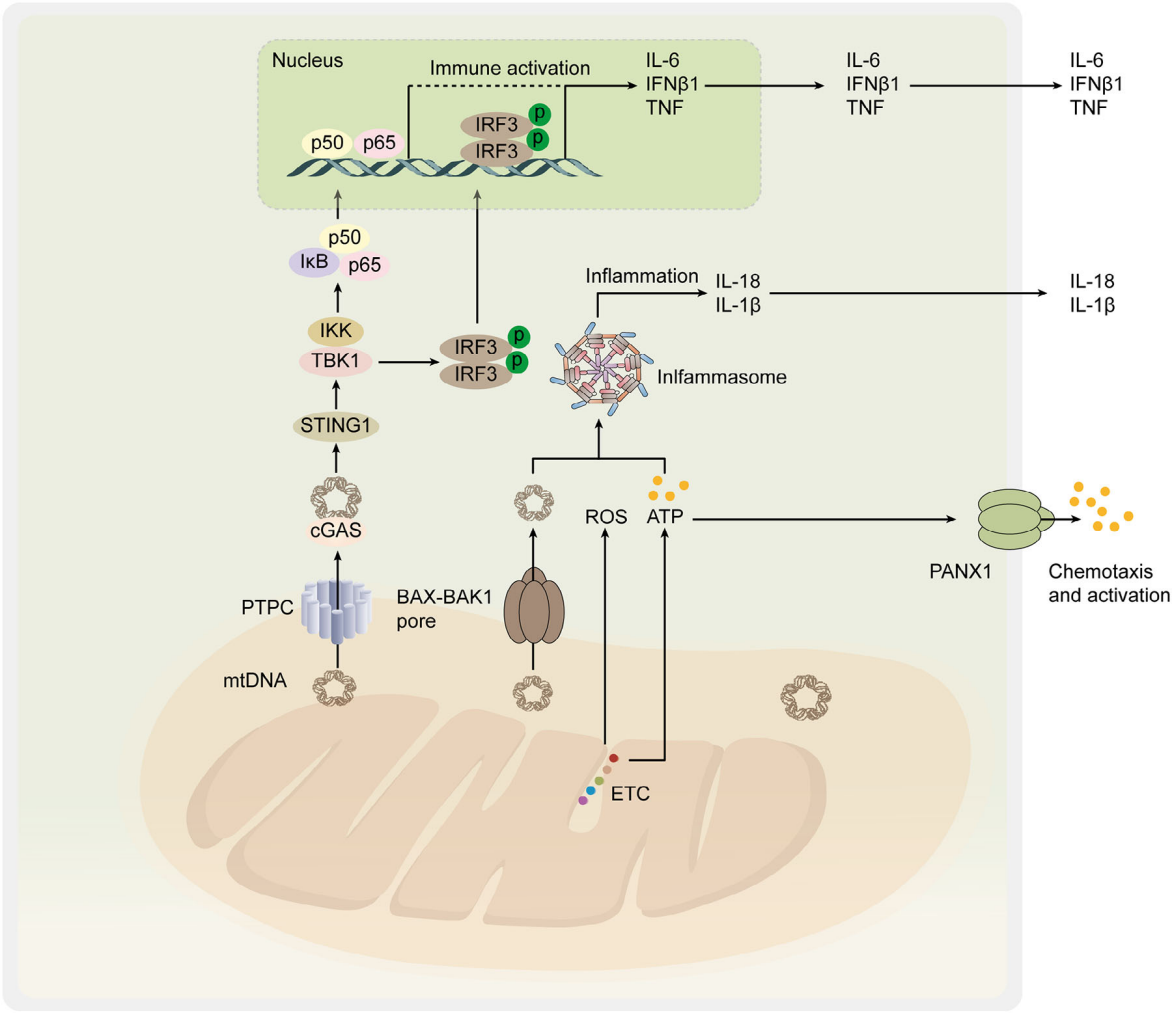
Mitochondrial DAMP signaling mediates inflammatory responses. Its principal mechanism involves the release of mitochondrial components and byproducts arising from mitochondrial dysfunction or cell death, which accumulate intracellularly or in the extracellular space and initiate inflammation. mtDNA can exit via BAX–BAK1 pores or the PTPC, subsequently acting as a potent activator of cGAS. Once cGAS is activated, STING1 signaling is triggered, leading to the production of cytokines such as IFNβ1, IL‐6, and TNF. Furthermore, under conditions of mitochondrial impairment, mtDNA and ROS released from mitochondria can promote IL‐1β and IL‐18 secretion through inflammasome pathways. ETC function may also modulate intracellular ATP availability via PCr, thereby influencing inflammasome activation independently of ROS. Cells undergoing death release ATP through lysosomal exocytosis and PANX1 channels; this ATP subsequently binds purinergic receptors to regulate the chemotaxis and immunostimulatory activity of APCs. *Abbreviations*: mtDNA, mitochondrial DNA; PTPC, permeability transition pore complex; cGAS, cyclic GMP–AMP synthase; IFNβ1, interferon‐β1; TNF, tumor necrosis factor; ETC, electron transport chain; PCr, phosphocreatine; PANX1, pannexin 1; APCs, antigen‐presenting cells.

In addition to being responsible for energy metabolism and the regulation of apoptosis, mitochondria also play a crucial role in the inflammatory response. Due to their origin from ancient bacteria and possessing a double‐membrane structure, when stress or death signals disrupt the integrity of their membranes, mtDNA, RNA, and related molecules (mtDAMPs) will be released and recognized by various pattern recognition receptors such as cGAS–STING, NLRP3, AIM2, TLR9, and RIG‐I, thus triggering different types of immune and inflammatory responses (Figure 5). Especially in the context of cardiac IRI, inflammation is closely linked to mitochondrial function [233, 234]. Inflammatory responses can lead to mitochondrial damage, while impaired mitochondria can release mtDNA and ROS, which can activate inflammatory reactions. Furthermore, ROS can also trigger inflammatory damage, creating a vicious cycle that exacerbates heart injury [99]. Some studies suggest that regulating mitochondrial function can influence the inflammatory response and mitigate cardiac IRI. For instance, certain antioxidants and mitochondrial protectants can reduce mitochondrial oxidative stress, thus reducing the severity of the inflammatory response [235, 236]. Additionally, by inhibiting excessive inflammatory responses, mitochondria can also be protected from damage. In the complex mechanisms of myocardial IRI, the close relationship between inflammation and mitochondrial function has become a focus of research. Recent studies have further elucidated the critical role of caspase‐8 activation and mitochondrial ROS production in IRI. During I/R, the inflammatory response, particularly the release of TNF‐α, activates caspase‐8. This activation not only initiates the apoptotic pathway but also leads to excessive mitochondrial ROS production. Elevated ROS levels impair the function of the ryanodine receptor (RyR2), resulting in abnormal calcium release and exacerbating myocardial tissue damage. Additionally, mitochondrial ROS promotes intracellular calcium overload and disrupts cellular homeostasis, further aggravating myocardial injury and functional impairment. Experimental data demonstrate that stabilizing RyR2 or inhibiting caspase‐8 activity significantly reduces ROS generation, thereby effectively protecting cardiac tissue. These findings suggest that targeting mitochondrial stress responses represents a promising therapeutic strategy for preventing I/R‐induced cardiac injury [237]. Another study revealed the pivotal role of sarcoplasmic/ER Ca^2^⁺‐ATPase (SERCA) in regulating mitochondrial function and suppressing inflammation. By enhancing SERCA activity through gene overexpression, researchers significantly improved MQC, including promoting mitochondrial fusion and autophagy, both of which are critical for maintaining cellular homeostasis. SERCA overexpression also modulated intracellular calcium balance, preserved mitochondrial function, and reduced ROS production associated with IRI. The findings demonstrated that maintaining mitochondrial health effectively decreased the expression of inflammatory markers and alleviated microvascular damage. This study underscores the central role of mitochondria in cardiac stress responses, particularly in controlling intracellular ROS levels and calcium homeostasis [238].

Overall, the interplay between inflammation, mitochondrial function, and cardiac IRI is highly intricate and interdependent, collectively shaping the processes of myocardial damage and repair. A deeper understanding of these mechanisms is critical for developing more effective therapeutic strategies to alleviate patient symptoms and enhance cardiac function. These studies not only provide significant insights into the pathophysiology of cardiac diseases but also lay a solid foundation for the development of novel drugs and treatment approaches, holding substantial potential for clinical translation and application.

### 4.4 Mitochondrial Biogenesis

**FIGURE 6:**
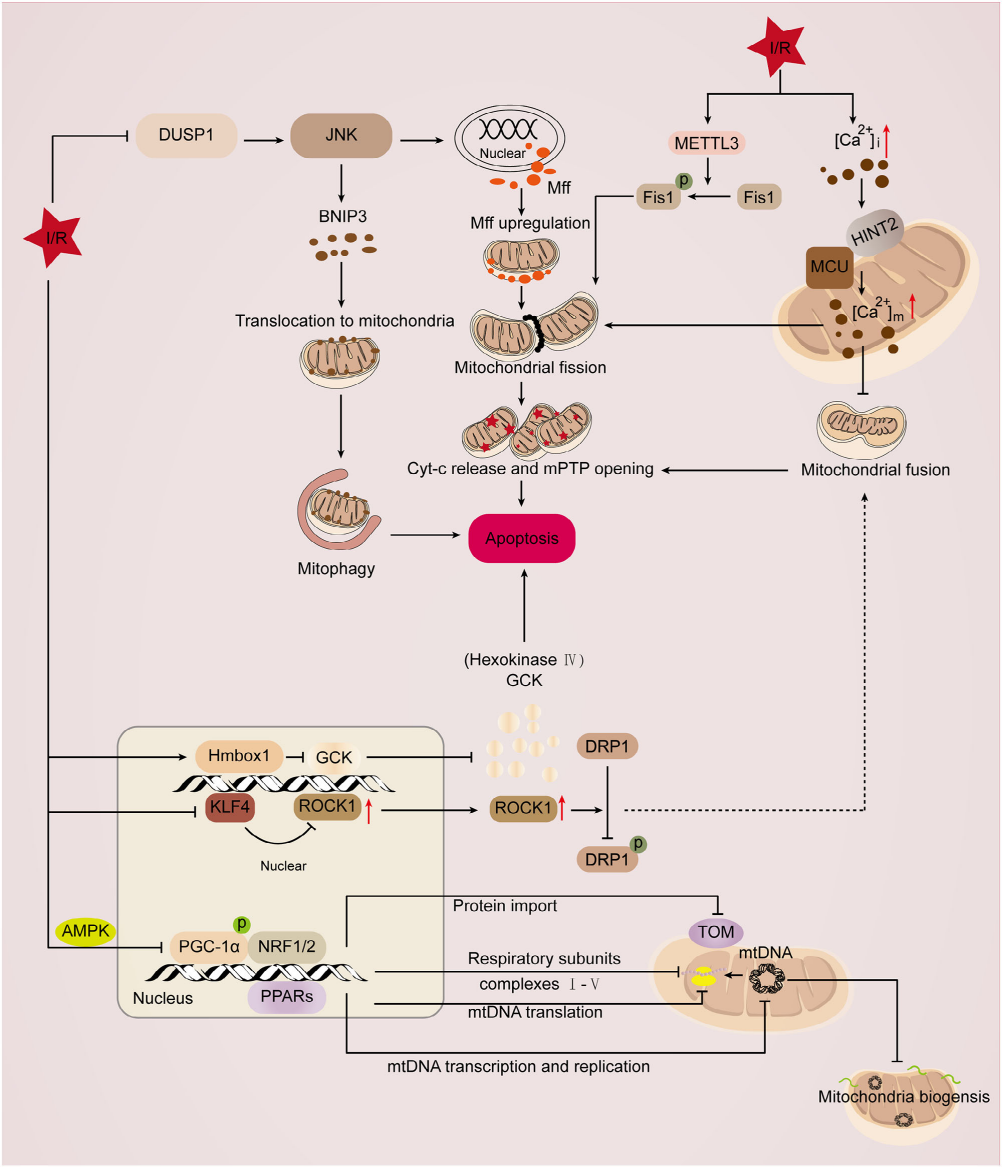
Regulation of mitochondrial biogenesis during I/R. I/R‐induced DUSP1 deficiency promotes the activation of JNK, which upregulates the expression of the Mff. Elevated levels of Mff are associated with increased mitochondrial fission and apoptosis. Additionally, the absence of DUSP1 amplifies BNIP3 phosphorylation activation via JNK, leading to enhanced mitophagy. The increased mitophagy markedly depletes mitochondrial quality, resulting in mitochondrial metabolic dysfunction. METTL3 deficiency can interfere with DNA‐PKcs phosphorylation, thereby preventing the downstream activation of Fis1 and subsequently inhibiting pathological mitochondrial fission. This ultimately improves cardiac function. Ca^2+^ overload is a key initiator of mitochondrial fission. HINT2 regulates the MCU complex by directly interacting with MCU in cardiac microvascular endothelial cells, thereby inhibiting Ca^2+^ overload. Overexpression of HINT2 suppresses the MCU complex‐induced mitochondrial calcium overload, preventing mitochondrial fission and apoptosis pathways, and thus alleviates cardiac microvascular ischemia–reperfusion injury. Under pathological conditions, the expression of Hmbox1 is upregulated. Inhibition of Hmbox1 activates the Akt/mTOR/P70S6K pathway and increases the transcription of GCK, leading to reduced cardiomyocyte apoptosis and improved mitochondrial respiration and glycolysis. This ultimately protects the heart. KLF4 deficiency can affect the expression of ROCK1 at the transcriptional level, thereby inducing DRP1‐mediated mitochondrial fission and ultimately exacerbating myocardial injury. Under pathological conditions, the process of mitochondrial biogenesis involves the inhibition of the interaction between PGC‐1α and Nrf1/2, PPARs, and suppresses the maintenance of mitochondrial oxidative phosphorylation function, inhibits the synthesis and import of nuclear gene‐encoded mitochondrial proteins, as well as the transcription, replication, and translation of mitochondrial DNA, thereby exacerbating damage. *Abbreviations*: DUSP1, dual specificity phosphatase 1; JNK, c‐Jun N‐terminal kinase; Mff, mitochondrial fission factor; BNIP3, BCL2 interacting protein 3; METTL3, methyltransferase like 3; DNA‐PKcs, DNA‐dependent protein kinase catalytic subunit; Fis1, fission 1; HINT2, histidine triad nucleotide binding protein 2; MCU, mitochondrial calcium uniporter; Hmbox1, heme oxygenase 1; Akt/mTOR/P70S6K: protein kinase B/mechanistic target of rapamycin/ribosomal protein S6 kinase beta‐1; GCK, glucokinase; KLF4, Krüppel‐like factor 4; ROCK1, Rho‐associated protein kinase 1; DRP1, dynamin‐related protein 1; PPARs, peroxisome proliferators‐activated receptors; TOM, translocator of the outer mitochondrial membrane; PGC1‐α, peroxisome proliferator‐activated receptor γ coactivator 1‐α.

Mitochondrial biogenesis, defined as the generation of new mitochondrial mass and replication of mtDNA via proliferation of existing mitochondria, is a key component of MQC [239]. The transcriptional coactivator PGC‐1α is a central regulator of this process, acting through downstream transcription factors such as NRF1, NRF2, PPAR‐α, steroid hormone receptors, and transcriptional repressors (Figure 6) [240, 241, 242, 243, 244, 245, 246]. Mitochondrial biogenesis plays a crucial role in IRI [247]. During myocardial IRI, mitochondrial biogenesis plays a critical compensatory role. Ischemic heart disease leads to mitochondrial dysfunction due to insufficient oxygen supply, while reperfusion exacerbates oxidative stress and further damages mitochondria. To mitigate this, cardiomyocytes activate mitochondrial biogenesis to restore mitochondrial function and sustain cellular energy metabolism. Pharmacological and natural compounds targeting biogenesis pathways, particularly via PGC‐1α, have demonstrated cardioprotective effects [10, 248]. For instance, ginsenoside Rd has potential benefits in the treatment of heart diseases. Specifically, Rd improves cardiac function and alleviates cardiac pathological changes in heart failure patients by modulating the interaction between adipocytes and myocardial cells. The mechanism of action involves promoting the secretion of omentin in adipocytes through the TBK1–AMPK signaling pathway, which in turn enhances mitochondrial biogenesis function and reduces cardiac ischemic injury. This research provides new insights into the treatment of heart failure, offering a novel therapeutic mechanism and potential drugs for the condition [249]. The combined treatment of nicotinamide mononucleotide (NMN) and MT was investigated for its effects on heart IRI in elderly rats. The study found that NMN/MT combination therapy significantly reduced the release of CK‐MB in the hearts of elderly rats after reperfusion. This protection was achieved through the regulation of multiple signaling pathways, including SIRT1/PGC‐1α/Nrf1/TFAM, mitochondrial fission/fusion, autophagy, and microRNA‐499 expression. The results of this research suggest that NMN/MT combination therapy can alleviate the burden of heart IRI in elderly patients, offering a potential therapeutic strategy for improving the cardiac health of the elderly population [250]. Dihydromyricetin (DHM) can reduce the area of MI, improve cardiac function, alleviate mitochondrial dysfunction, and reduce oxidative stress levels. Furthermore, the beneficial effects of DHM depend on the upregulation of Sirt3. Research findings indicate that DHM improves mitochondrial function and alleviates oxidative stress by increasing Sirt3 levels, offering a potential therapeutic pathway for patients with heart IRI [251]. These drugs and natural compounds may provide cardiac protection by increasing mitochondrial numbers and improving mitochondrial function, thereby reducing the severity of heart IRI. Therefore, mitochondrial biogenesis plays a crucial protective role in heart I/R, contributing to the maintenance of cardiac health. Connexin 43 (Cx43) is a product of the GJA1 gene and serves as a gap junction protein that facilitates intercellular communication among cardiac muscle cells. Research has indicated that under conditions of ischemia and IRI, the expression levels of endogenous GJA1‐20k protein in the heart are upregulated. This GJA1‐20k protein localizes to cardiac mitochondria and interacts with the mitochondrial outer membrane. Importantly, studies have found that AAV9‐mediated delivery of the GJA1‐20k gene can increase mitochondrial biogenesis in mouse hearts while simultaneously reducing mitochondrial membrane potential, respiratory function, and ROS production. This action allows GJA1‐20k to promote a protective mitochondrial phenotype similar to ischemic preconditioning. In summary, GJA1‐20k is an endogenous stress response protein that operates by inducing mitochondrial biogenesis and regulating metabolic status, thus preemptively protecting the heart from IRI. The introduction of exogenous GJA1‐20k may offer a potential therapeutic strategy for patients at risk of ischemic injury [252]. The United States Food and Drug Administration‐approved HDAC inhibitor drug SAHA (Vorinostat) has been shown to induce autophagy in cardiac myocytes and reduce the extent of myocardial IRI. Before simulating I/R, SAHA preconditioning significantly increased the mtDNA content and mitochondrial mass of cardiac myocytes. In vivo, IRI led to a more than 50% decrease in mtDNA content in the mouse heart's border zone. However, SAHA preconditioning and reperfusion treatment alone restored mtDNA content and mitochondrial mass to normal levels. Furthermore, cardiac myocyte preconditioning with SAHA resulted in a fourfold reduction in the loss of mitochondrial membrane potential induced by I/R and a 25–40% decrease in cytoplasmic ROS levels. However, the loss of ATG7 function in cardiac myocytes or the mouse heart abolished the protective effects of SAHA on ROS levels, mitochondrial membrane potential, mtDNA levels, and mitochondrial mass. Last, SAHA induced the expression of the PGC‐1α gene in NRVMs (neonatal rat ventricular myocytes) and the hearts subjected to I/R. Deletion of PGC‐1α eliminated the mitochondrial protective effects of SAHA. These findings suggest that SAHA has a protective effect on mitochondrial function and reduces IRI by inducing autophagy, preserving mtDNA, and improving mitochondrial quality. Additionally, its protective effects are dependent on ATG7 function and PGC‐1α expression [253]. The authors collected left ventricular tissue from patients with advanced heart failure and nonheart failure hearts (23 cases and 19 cases, respectively). In cases where citrate synthase activity moderately decreased, mtDNA content in heart failure patients decreased by more than 40% (*p* < 0.05). This was accompanied by a reduction in protein encoded by mtDNA (25–80%) at both the mRNA and protein levels (*p* < 0.05). The mRNA levels of PGC‐1α/β and PRC (PGC‐1‐related coactivator) remained unchanged, while PGC‐1α protein increased by 58% in heart failure patients. Among the PGC‐1 coactivator targets, the expression of estrogen‐related receptor alpha and its downstream genes decreased by up to 50%, while PPAR‐α and its downstream genes remained unchanged in heart failure patients. The formation of the mtDNA‐loop was normal, but D‐loop expansion, a part of the mtDNA replication process, decreased by 75%. Additionally, DNA oxidative damage increased by 50% in heart failure patients [254]. As evidenced by the reduction in mtDNA replication and mtDNA depletion, mitochondrial biogenesis is severely impaired in human heart failure. These defects are unrelated to the downregulation of PGC‐1 expression, suggesting a novel mechanism for mitochondrial dysfunction in heart failure. The study investigated mitochondrial autophagy and mitochondrial biogenesis in patients undergoing cardiac surgery involving cardiopulmonary bypass. The research revealed signs of mitochondrial autophagy in postoperative samples, characterized by a decrease in autophagy adapter levels, a reduction in the long form of Opa1, and Parkin translocation to mitochondrial fractions. Simultaneously, mtDNA damage increased, but surprisingly, mitochondrial biogenesis also significantly increased, manifested by an increase in several mitochondrial markers and the mtDNA/nucDNA ratio. The study further found that mitochondrial biogenesis appears to be independent of transcription and may be driven by an increase in the translation of existing mRNA. These results suggest that both mitochondrial autophagy and mitochondrial biogenesis occur during cardiac surgery, possibly in response to I/R stress during the procedure. Further research into the regulation of mitochondrial turnover and mitigating mtDNA damage may help improve the prognosis of heart disease patients after I/R [255].

## 5 Conclusion and Outlook

MQC plays a crucial role in maintaining cellular homeostasis and tissue function, and its dysfunction is closely associated with the pathogenesis of various human diseases, including myocardial IRI, cerebral ischemia–hypoxia injury, renal diseases, inflammatory disorders, and neurodegenerative diseases. MQC primarily operates through coordinated mechanisms involving mitochondrial biogenesis, mitochondrial dynamics (fusion and fission), and mitophagy, which precisely regulate mitochondrial quantity, quality, and function. These mechanisms facilitate timely clearance of damaged mitochondria, reduce ROS production, and consequently inhibit various forms of cell death, including apoptosis, necrosis, pyroptosis, and ferroptosis, thereby effectively mitigating tissue injury. In recent years, natural compounds such as quercetin and resveratrol, small molecule compounds like SS‐31 and nicotinamide, and novel mitochondria‐targeted delivery systems have demonstrated significant protective effects in diverse disease models, indicating promising clinical translational potential for MQC‐targeted strategies.

However, several challenges remain in MQC research regarding clinical translation: (1) The heterogeneity of MQC across various disease models, tissues, and cell types has yet to be fully elucidated. For instance, mitochondrial dynamics and mitophagy mechanisms differ significantly between cardiomyocytes and neuronal cells under pathological conditions. Future studies must clarify cell type‐specific molecular mechanisms of MQC regulation to enhance precision in MQC‐targeted therapies. (2) Complex and intersecting signaling networks exist among mitochondrial biogenesis, mitophagy, and dynamics, which may exhibit varying degrees of activation or inhibition at different stages of disease progression. Future research needs to thoroughly investigate the interactions within these signaling pathways and develop systematic models to define optimal coordination strategies of different MQC mechanisms under specific pathological contexts. (3) The safety, specificity, and stability of MQC‐targeted drugs in clinical research require further improvement. Current mitochondria‐targeted antioxidants and modulators of mitochondrial dynamics have limitations, including narrow therapeutic windows, potential adverse reactions, off‐target effects, and individual variability in therapeutic responses. Enhancing the precision of drug design, specificity of target selection, and optimization of dosage and timing represent critical directions for future drug development. (4) The absence of precise, real‐time, and noninvasive tools for assessing mitochondrial function and ROS dynamics remains a major obstacle in advancing mechanistic studies of MQC and hindering its clinical translation. To overcome these limitations, future efforts should focus on the development of real‐time imaging technologies, single‐cell metabolic profiling platforms, and nanotechnology‐based detection systems with high sensitivity and specificity. These innovations are expected to enable more accurate monitoring of mitochondrial behavior under physiological and pathological conditions, thereby driving the application of MQC research in precision medicine.

Future research on MQC should prioritize the identification and functional characterization of key regulatory molecules and signaling hubs within MQC networks. Efforts are also needed to develop modulators that are more selective, effective, and clinically safe. In addition, exploring personalized therapeutic strategies targeting MQC may enhance treatment outcomes across heterogeneous disease populations. To facilitate clinical translation, a multidisciplinary approach integrating basic research, disease modeling, and translational studies is essential. Furthermore, the application of multiomics technologies and advanced computational tools, including artificial intelligence, may enable the construction of precision MQC regulatory models. Such models hold promise for improving the specificity and efficacy of MQC‐targeted interventions, ultimately contributing to better strategies for disease prevention and treatment.

## Author Contributions

Ye Lin: writing—review and editing, writing—original draft, validation, resources, methodology, and investigation. Xinzhi Fu: writing—review and editing, validation, methodology, and formal analysis. Qi Li: writing—review and editing, supervision, funding acquisition, and conceptualization. All authors have read and approved the final manuscript.

## Conflicts of Interest

The authors declare no conflicts of interest.

## Data Availability

Data will be made available on request.
